# Sequential deregulation of histone marks, chromatin accessibility and gene expression in response to PROTAC-induced degradation of ASH2L

**DOI:** 10.1038/s41598-023-49284-x

**Published:** 2023-12-19

**Authors:** Mirna Barsoum, Roksaneh Sayadi-Boroujeni, Alexander T. Stenzel, Philip Bussmann, Juliane Lüscher-Firzlaff, Bernhard Lüscher

**Affiliations:** 1 Institute of Biochemistry and Molecular Biology, Faculty of Medicine, RWTH Aachen University, Pauwelsstrasse 30, 52074 Aachen, Germany; 2 Present Address: Bayer AG, Crop Science Division, R&D, Pest Control, 40789 Monheim am Rhein, Germany; 3 Present Address: Institute of Human Genetics, Faculty of Medicine, University of Bonn, Venusberg-Campus 1, 53127 Bonn, Germany

**Keywords:** Epigenetics, Chromatin, Histone post-translational modifications, Molecular biology, Transcription

## Abstract

The trithorax protein ASH2L is essential for organismal and tissue development. As a subunit of COMPASS/KMT2 complexes, ASH2L is necessary for methylation of histone H3 lysine 4 (H3K4). Mono- and tri-methylation at this site mark active enhancers and promoters, respectively, although the functional relevance of H3K4 methylation is only partially understood. ASH2L has a long half-life, which results in a slow decrease upon knockout. This has made it difficult to define direct consequences. To overcome this limitation, we employed a PROTAC system to rapidly degrade ASH2L and address direct effects. ASH2L loss resulted in inhibition of proliferation of mouse embryo fibroblasts. Shortly after ASH2L degradation H3K4me3 decreased with its half-life varying between promoters. Subsequently, H3K4me1 increased at promoters and decreased at some enhancers. H3K27ac and H3K27me3, histone marks closely linked to H3K4 methylation, were affected with considerable delay. In parallel, chromatin compaction increased at promoters. Of note, nascent gene transcription was not affected early but overall RNA expression was deregulated late after ASH2L loss. Together, these findings suggest that downstream effects are ordered but relatively slow, despite the rapid loss of ASH2L and inactivation of KMT2 complexes. It appears that the systems that control gene transcription are well buffered and strong effects are only beginning to unfold after considerable delay.

## Introduction

In eukaryotic cells, the genome is organized as chromatin to control the access to and the use of the DNA in processes such as transcription, replication and repair. The smallest unit of chromatin is the nucleosome, which is composed of the four core histones H2A, H2B, H3 and H4 or variants thereof, and 147 base pairs of DNA^1^. Linker DNA of various length connects individual nucleosomes. Higher order chromatin organization involves loops, topologically associated domains or TADs, and A/B compartments, defined at least in part by factors such as CTCF and cohesin^2,3^. The core of nucleosomes is formed by the globular regions of histones, while the N-terminal tails protrude, which makes them accessible to a wide range of post-translational modifications (PTMs)^4,5^. These are the product of a large panel of enzymes that include writers such as methyltransferases and acetyltransferases and the corresponding erasers, thereby controlling access to nucleosomes, DNA and more general chromatin. Sequence-specific transcription factors are key to direct and assemble these enzymes to distinct regions in chromatin and to coordinate the regulation of gene transcription^6,7^.

Reversible methylation of histone H3 at lysine 4 (H3K4) has been linked to gene transcription^8–11^. Lysines can be mono-, di- or tri-methylated (Kme1-3), thereby altering the spectrum of reader molecules that are able to interact. While H3K4me1 is primarily located at enhancers, H3K4me3 is a mark of open chromatin at promoters. Together with H3K27 acetylation (H3K27ac), these marks define active enhancers and promoters, respectively^12–15^. In contrast, H3K4me3 in combination with H3K27me3 establish bivalent chromatin, which is linked to poised promoters^16,17^. Multiple readers have been described, which are thought to convey information encoded in H3K4 methylation, whose effects include chromatin remodeling, RNA polymerase (RNAPII) loading, and H3K4 methylation amplification^18,19^.

H3K4 methylation is catalyzed by COMPASS (complex of proteins associated with Set1)^9–11^. This complex, originally defined in yeast^20^, exists in 6 versions in mammals, defined by 6 different catalytic subunits, KMT2A-D, F and G (MLL1-4, SET1A and B, respectively). All 6 versions contain a core complex of 4 proteins, WDR5, RBBP5, ASH2L and 2 copies of DPY30, the so-called WRAD complex, which is necessary for efficient methyltransferase activity^21–26^. Additional subunits have been described that are specific for certain KMT2 complexes^10,27^. As far as studied, all the subunits of KMT2 complexes are essential for proper cell functioning, particularly the WRAD subunits, as their deletion results in distinct developmental defects in model organisms^28–37^. This poses challenges for WRAD subunit analyses as broad effects on chromatin and gene expression are expected and observed, resulting in complex phenotypes. Also, many of the subunits are mutated or their expression deregulated in diseases, including cancer, neurodegeneration and complex syndromes^27,38,39^. Of note is that the WRAD complex interacts with multiple sequence-specific transcription factors and thus appears to be important for recruiting COMPASS-like complexes to specific sites in chromatin^27^. In addition, these complexes interact with the RNAPII complex and with CpG islands^11,40^. Thus, the different KMT2 complexes are assembled from common and selective subunits that are important for optimal catalytic activity and chromatin localization.

ASH2L is necessary for organismal development^35,41^. Moreover, we have previously observed that deletion of *Ash2l* in the hematopoietic system prevents proliferation and differentiation of hematopoietic cells, ultimately resulting in the death of the animals^29^. Of note is that the loss of Dpy30 provokes a very similar phenotype^28,42^. The KO of either *Ash2l* or *Dpy30* results in a strong decrease in both bone marrow and peripheral hematopoietic cells, and a proliferation and differentiation defect of multi-potent progenitor cells. This suggests that the main functions of Ash2l and Dpy30 are associated with the WRAD complex and thus with KMT2 complexes. We identified ASH2L as an interaction partner of the oncoprotein MYC^43,44^, indicating that this transcription factor can recruit KMT2 complexes. Indeed, sequential chromatin immunoprecipitation (ChIP) experiments documented that the two proteins can co-localize to known MYC response elements and that binding of MYC is associated with increased H3K4me3^44^. Mechanistically, downregulation or loss of ASH2L provokes a decrease of H3K4me3 at promoters, associated with altered gene transcription^29,44–47^. Somewhat counterintuitive, the loss of Ash2l and the decrease in H3K4me3 at promoters, both linked positively to gene expression, cause both repression and activation of gene transcription. We have argued previously that activation might well be a secondary effect, for example when a transcriptional repressor is no longer expressed^46,47^. This might be particularly relevant in experimental settings that are characterized by a slow response such as upon applying siRNA or using classical recombination (see also below). Together, these studies suggested that in cells ASH2L is necessary for efficient H3K4me3, affecting gene expression, findings that are consistent with a determining function of H3K4me3 for promoter activity.

Our previous work, based on efficient and fast recombination of the floxed *Ash2l* alleles, was hampered due to the long half-life of ASH2L proteins, associated with a slow development of phenotypes. For example, in mouse embryo fibroblasts (MEFs) substantial effects on H3K4 methylation and gene expression were seen only after several days upon loss of Ash2l^46,47^. Similarly, the manifestation on cell proliferation inhibition, cell cycle arrest and induction of senescence occurred after 5 days or later. Thus, due to the sluggishness of the system, it has been difficult to separate and distinguish primary from secondary and even tertiary effects. Therefore, we employed a system, in which we were able to deregulate ASH2L rapidly. We generated an FKBP-ASH2L fusion protein that is sensitive to a proteolysis targeting chimera (PROTAC). FKBP-ASH2L was expressed in MEF cells with floxed *Ash2l* alleles, the endogenous alleles were deleted, rendering the cells dependent on the introduced fusion protein. The PROTAC dTAG-13 binds to FKBP and Cereblon, a subunit of an E3 ligase, and destines FKBP fusion proteins for degradation^48^. The rapid loss of FKBP-ASH2L inhibits cell proliferation and promotes a consecutive modulation of histone marks at both H3K4 and H3K27, alters the accessibility of chromatin, and deregulates gene expression.

## Results

### Loss of ASH2L prevents cell proliferation

**Figure 1:**
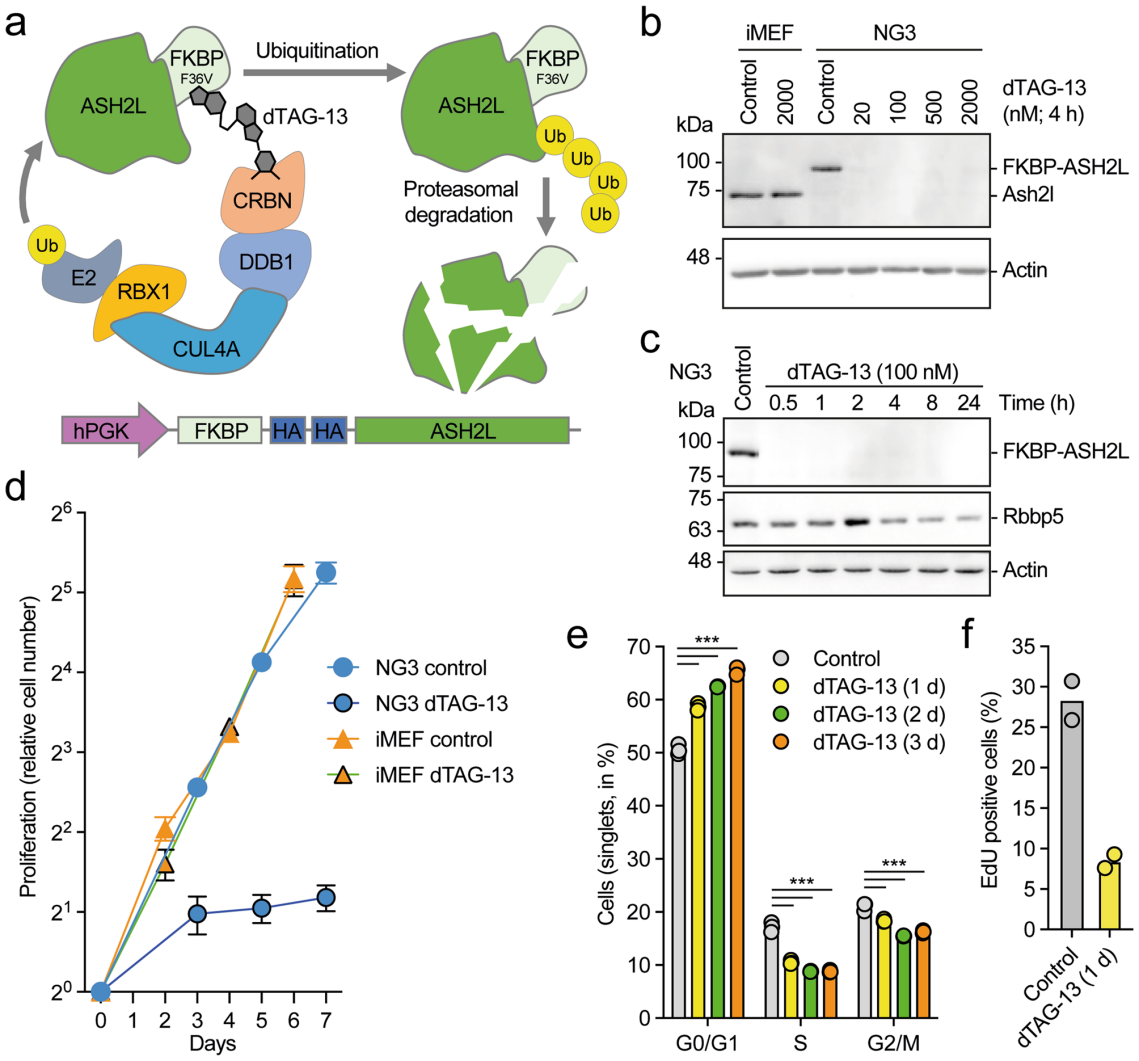
PROTAC-induced rapid degradation of ASH2L inhibits cell proliferation. (**a**) Schematic summary of dTAG-13-mediated degradation of FKBP-HA_2_-ASH2L fusion proteins. (**b**) iMEF control cells and NG3 cells were treated with dTAG-13 as indicated. Total cell lysates were analyzed for Ash2l and FKBP-HA_2_-ASH2L expression using ASH2L/Ash2l selective antibodies. Actin staining was used as loading control. (**c**) As in panel B with dTAG-13 treatment for the indicated times. In addition to FKBP-HA_2_-ASH2L and actin, Rbbp5 was visualized. (**d**) iMEF control cells and FKBP-HA_2_-ASH2L fusion proteins expressing NG3 cells were treated with or without dTAG13 from day 0. Cells were counted at the indicated time points. Measurement were in triplicates with three biological replicates. Indicated are relative mean values ± SEM. (**e**) NG3 cells were treated with dTAG-13 (100 nM) for the indicated times. The cells were fixed and stained with Hoechst 33258 and analyzed by flow cytometry. Mean values of three measurements in triplicates are displayed. (***p ≤ 0.001). (**f**) NG3 cells were treated with or without dTAG-13 (100 nM) for 24 h. During the final three hours, the cells were incubated with EdU, fixed and stained with AF488 azide. Mean values of two flow cytometry measurements in duplicates are shown.

We have studied the molecular and cellular consequences of Ash2l loss in mouse embryo fibroblasts (MEFs) with floxed *Ash2l* alleles and an inducible Cre-ER recombinase (iMEF-*Ash2l*^*fl/fl*^-Cre-ER). While the knockout of *Ash2l* was rapid, the downstream effects, including the decrease in promoter-associated H3K4me3, altered gene expression, and cell cycle and proliferation arrest, were slow likely due to the long half-life of Ash2l^43,46,47^. Thus, to define direct consequences of the loss of Ash2l and to distinguish these from secondary and further downstream effects has been difficult. To overcome this, we implemented a PROTAC system (summarized schematically in Fig. 1a). We generated a plasmid that expresses FKBP-F36V fused through a linker with 2 HA-tags to human ASH2L. FKBP-F36V is a mutant version of the prolyl isomerase FKBP12, which has been engineered to accommodate a ligand that cannot bind to the wild-type protein^48–50^. FKBP-HA_2_-ASH2L can be tied to Cereblon (CRBN), a component of an E3 ubiquitin ligase complex, using the heterobifunctional compound dTAG-13^48^. Thus, the proximity of the proteins induced by dTAG-13 promotes poly-ubiquitination of the FKBP-tagged fusion protein and subsequently results in its degradation. The construct expressing FKBP-HA_2_-ASH2L was introduced into the iMEF-*Ash2l*^*fl/fl*^-Cre-ER cells. Then, exon 4 of the endogenous *Ash2l* was deleted upon activation of Cre-ER and individual clones selected (Supplementary Fig. S1a). The RNA corresponding to exon 4 of *Ash2l* could no longer be detected in these cells (Supplementary Fig. S1b)^47^.

The expression of the endogenous Ash2l and the FKBP-HA_2_-ASH2L fusion protein was analyzed in response to dTAG-13 (Fig. 1a). We established three clones, NG3, ND10 and NB5 (Supplementary Fig. S1a), in which the endogenous Ash2l could no longer be detected (Fig. 1b and Supplementary Fig. S1c, d). The expression of the introduced FKBP-HA_2_-ASH2L was comparable to the endogenous Ash2l (Fig. 1b). In all three clones the FKBP-HA_2_-ASH2L protein was sensitive to dTAG-13 and the protein was degraded efficiently by 30 min (Fig. 1b,c, Supplementary Fig. S1c,d). Titration experiments of control lysates suggested that less than 1% of FKBP-HA_2_-ASH2L remained after 1 h of dTAG-13 treatment (Supplementary Fig. S1e). Further studies were predominantly performed with NG3 cells.

The loss of Ash2l results in inhibition of proliferation and a block of cell cycle progression in both MEF cells and in hematopoietic multi-potent progenitors^29,47^. In the latter, human ASH2L rescues cell proliferation in tissue culture^29^. Similarly, the FKBP-HA_2_-ASH2L clones, in the absence of endogenous Ash2l, proliferated comparably to control iMEF cells (Fig. 1d and Supplementary Fig. S2a). These control cells were unaffected by dTAG-13 (Fig. 1d). In contrast, all three FKBP-HA_2_-ASH2L clones stopped proliferating rapidly, for example, clone NG3 cells doubled once initially and then cell numbers remained constant (Fig. 1d). Clones NB5 and ND10 were also inhibited (Supplementary Fig. S2a). A small increase in G0/G1 cells was noted over 3 days, but no distinct cell cycle arrest was observed (Fig. 1e), yet the incorporation of the thymidine analog 5-ethynyl-2′-deoxyuridine (EdU) decreased strongly within 24 h (Fig. 1f and Supplementary Fig. S2b). Thus, we did not observe a prominent accumulation of cells at a specific checkpoint. Rather, cells arrested throughout the cell cycle, including in S phase. It supports the notion that the loss of ASH2L generates dependencies on various factors that are relevant during different cell cycle phases, as discussed further below. A similar observation and conclusion were drawn from the analysis of our iMEFs cells upon *Ash2l* knockout^47^.

We did not observe any sign of apoptosis or senescence in the first 24 h. Also, neither p53 expression nor phosphorylation of H2A.X (γ-H2A.X) were induced as might occur upon altered chromatin organization or replication stalling and associated DNA damage (Supplementary Fig. S2c,d). For control, cells were treated with etoposide, which promoted p53 accumulation and H2A.X phosphorylation. Together, these findings reiterate that ASH2L is necessary for cell proliferation.

### ASH2L loss deregulates gene expression

**Figure 2:**
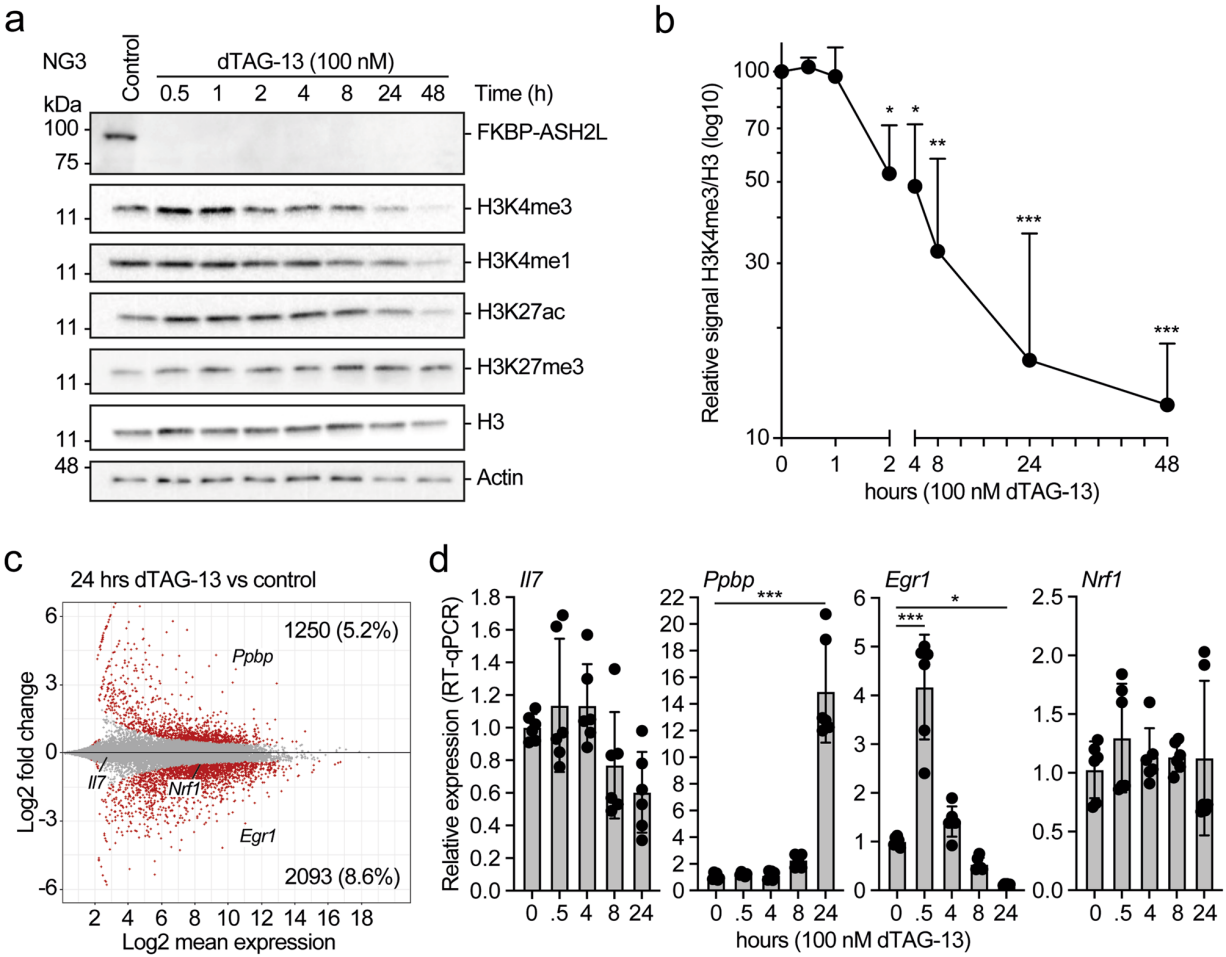
Decrease of H3K4me3 and altered gene expression upon loss of ASH2L. (**a**) NG3 cells were treated with dTAG-13 for the indicated times. Whole cell lysates were analyzed and the indicated antigens stained on Western blots using selective antibodies. (**b**) Quantification of H3K4me3 signals compared to total histone H3. Mean of three measurements with SD. (*p ≤ 0.05; ** ≤ 0.01; ***p ≤ 0.001). (**c**) NG3 cells were treated with or without dTAG-13 for 24 h. Whole RNA was extracted and analyzed using 3′mRNA-seq. Displayed is an MA plot. The number of genes that are significantly up- or down-regulated are indicated (red dots: q < 0.05). The data summarize two replicates normalized using ERCC spike-in RNA. (**d**) RT-qPCR analysis of gene expression in response to dTAG-13 treatment. Mean values and standard deviations of 5–6 measurements are displayed. (*p ≤ 0.05; ***p ≤ 0.001).

ASH2L is necessary for assembling the WRAD complex, which is required for efficient H3K4 methyltransferase activity of all six KMT2 enzymes and thus for global H3K4 methylation. Indeed, the loss of Ash2l in the iMEF system reduced H3K4 methylation, a process that took several days^46,47^. In the PROTAC system, a rapid decrease of H3K4me3 was measured (Fig. 2a and Supplementary Fig. S1c,d). Assuming that very little KMT2 catalytic activity remains, the decrease in H3K4me3 should depend on demethylases that have been described to remove methylation from H3K4, including KDM5 family members and LSD1^8,51^. Quantification of Western blots and comparing the H3K4me3 signals to total H3 revealed that until 1 h no decrease in methylation was measured. Subsequently, the decrease was rapid between 1 and 8 h and considerably slower until 48 h (Fig. 2b). At this time point, roughly 10% of H3K4me3 was remaining. The overall decrease in H3K4me1 was slower (Fig. 2a), possibly in part due to an increase in mono-methylation at promoters, as discussed below. Further, the sensitivity of H3K4me3 and H3K4me1 to the different demethylases may be distinct^8,51^. The decrease in H3K27ac was also slow, while H3K27me3 did not change (Fig. 2a). Thus, although FKBP-HA_2_-ASH2L was rapidly lost upon dTAG-13 treatment, the changes of the measured histone marks were considerably delayed.

The decrease of H3K4me3, a histone mark that is predominantly found at active promoters, is expected to alter gene transcription. Upon loss of FKBP-HA_2_-ASH2L, more than 3000 genes were deregulated by 24 h. The majority of genes showed reduced expression, but a substantial number of genes were upregulated (Fig. 2c, Supplementary Table S1; available in GEO under accession number GSE240987). These numbers were higher than what we had observed in the iMEF cells 5 days after *Ash2l* KO^47^. The changes measured were confirmed by RT-qPCR analyses of transcripts that were repressed, induced or remained unchanged (Fig. 2d). Of note is *Egr1*, a gene known to be induced upon various signaling processes, including growth factors, cytokines and different forms of stress^52,53^, which was initially stimulated in response to dTAG-13 treatment and degradation of FKBP-HA_2_-ASH2L. This suggested that inactivating KMT2 complexes resulted in rapid altered signaling and/or altered sensitivity to signals that control *Egr1* expression.

As expected from the strong inhibition of proliferation, many genes expressing cell cycle relevant proteins, including G1, G1-S, S and G2-M cyclins and replication factors, were downregulated (Supplementary Fig. S2e, Supplementary Table S1; available in GEO under accession number GSE240987). The expression of genes encoding the different Kmt2 methyltransferases, Wdr5, Rbbp5 and Dpy30 was not affected (Supplementary Table S1; available in GEO under accession number GSE240987). Consistent with this finding, the expression of Rbbp5 and Wdr5 was not altered, even after prolonged dTAG-13 treatment (Fig. 2a and Supplementary Figs. S1c,d, and S2f). Thus, it appears that targeting ASH2L does not result in the degradation of other WRAD complex components as might be expected from cross-poly-ubiquitination of the other subunits. It is possible that this occurs but once the existing complexes are depleted, FKBP-HA_2_-ASH2L might be targeted for degradation before efficient assembly into WRAD complexes can occur.

### Sequential changes in histone marks upon ASH2L loss

**Figure 3:**
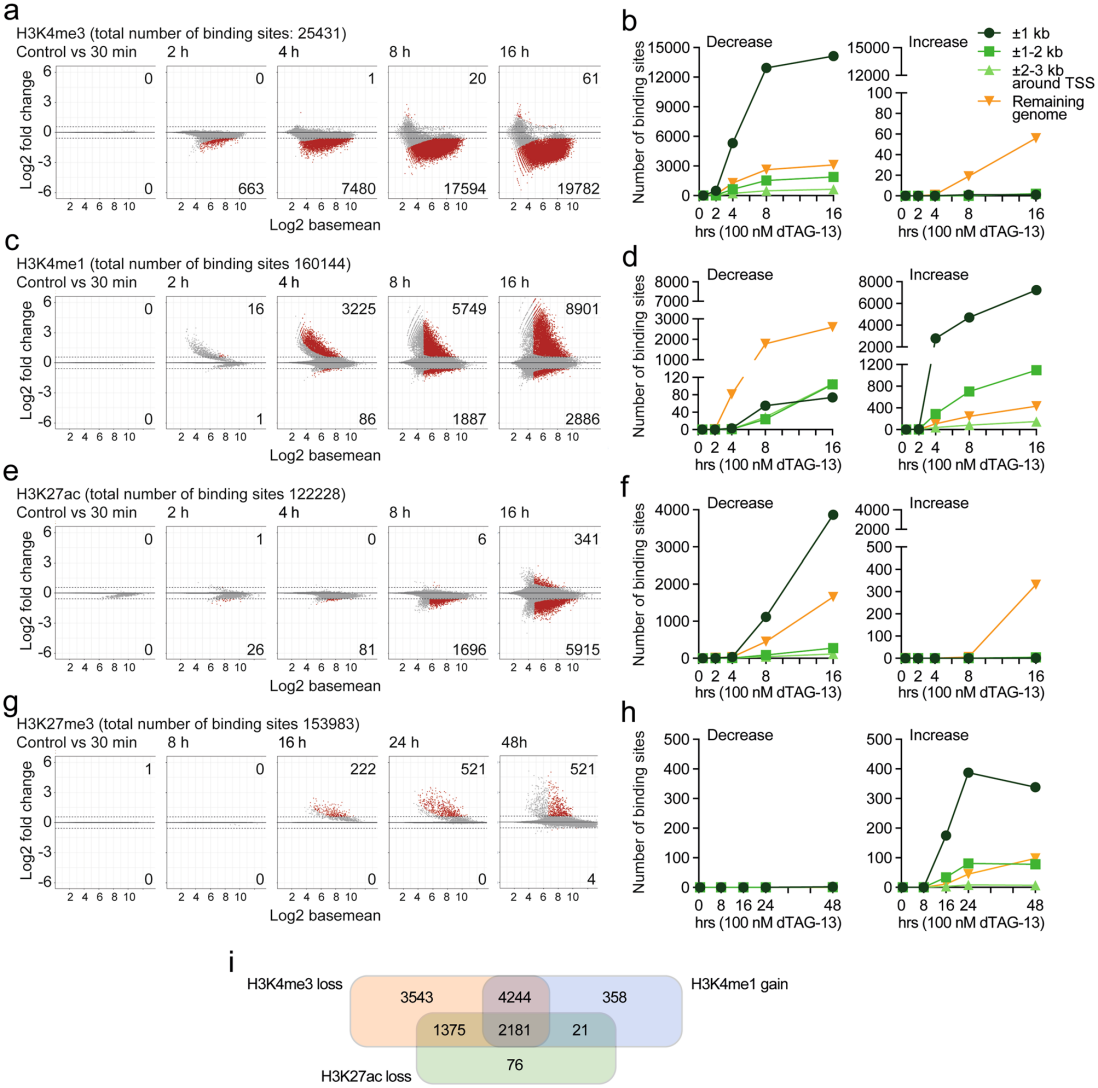
Promoter-associated decrease of H3K4me3 and increase of H3K4me1 upon loss of ASH2L. ChIP-seq experiments of the histone marks H3K4me3 (panels **a** and **b**), H3K4me1 (panels **c** and **d**), H3K27ac (panels **e** and **f**), and H3K27me3 (panels **g** and **h**) were performed on cells treated with dTAG-13 for the times indicated (2 biological replicates are summarized; NG3 cells). Panels (**a**, **c**, **e**, and **g**) display MA plots. Given are the total number of binding sites identified across all samples and the significantly increased and decreased sites (red dots: q < 0.05 and logFC > 0.58) upon loss of FKBP-HA_2_-ASH2L. Panels (**b**, **d**, **f**, and **h**) are summary graphs that detail the annotation of significantly changed sites to regions associated with transcriptional start sites (TSS) compared to the remaining genome. Panel (**i**) shows the overlap of promoters that lost H3K4me3, gained H3K4me1 and lost H3K27ac.

To further evaluate the consequences of FKBP-HA_2_-ASH2L loss, we performed ChIP-seq experiments. Essentially all FKBP-HA_2_-ASH2L binding sites (6444 sites identified across all samples, Supplementary Table S2a; available in GEO under accession number GSE240987), evaluated using HA-selective antibodies, showed considerably less signal within 1 h of dTAG-13 treatment (Supplementary Table S2a and b; available in GEO under accession number GSE240987, see also below). For example, FKBP-HA_2_-ASH2L could no longer be detected at the *Rspo2* and *Zfp503* promoters upon dTAG-13 treatment (Supplementary Fig. S3a). The H3K4 and H3K27 histone marks were measured in time course experiments upon loss of FKBP-HA_2_-ASH2L (Fig. 3, Supplementary Tables S3–S7; available in GEO under accession number GSE240987). The ChIP-seq experiments were performed in duplicates, correlation matrix heat maps demonstrated similarity (Supplementary Fig. S3b). We detected H3K4me3 signals at 25,431 sites, most of these near transcription start sites (TSS, Supplementary Table S3a; available in GEO under accession number GSE240987). The majority of H3K4me3 binding sites lost signal after 8 h with little further loss after 16 h (Fig. 3a, Supplementary Table S3b; available in GEO under accession number GSE240987). We rarely detected an increase in signal. Noticeable was that the downregulated sites were promoter-associated with a strong preference for the ± 1 kb window around TSSs (Fig. 3b). We analyzed the genes associated with the H3K4me3 sites that lost signal 2 h after the addition of dTAG-13 using Gene Ontology (GO) (Supplementary Fig. S3c). We noted terms linked to chromatin, replication and cell cycle. This is consistent with the unspecific cell cycle arrest described above and the effects on chromatin organization (see below). Despite the overall decrease in H3K4me1 signals, a substantial number of sites showed increased signals (Fig. 3c, Supplementary Table S4; available in GEO under accession number GSE240987). These were almost exclusively located at promoters, while sites with decreased signals were preferentially associated with non-promoter sites (Fig. 3d).

Methylation at H3K4 is connected to acetylation and methylation at H3K27^11,16,54^. Both global H3K27ac and H3K27me3 showed little difference over the first 24 h upon FKBP-HA_2_-ASH2L loss (Fig. 2a). H3K27ac decreased slightly by 48 h. The ChIP-seq analysis revealed more than 122,000 H3K27ac sites, of which roughly 5% showed a reduced signal after 16 h (Fig. 3e, Supplementary Table S5; available in GEO under accession number GSE240987). The majority of these were at promoters and more specifically within ± 1 kb of TSSs (Fig. 3f). Sites with increased signals were rare. The findings were verified using ChIP-qPCR. At two selected promoters a decrease of H3K4me3 and H3K27ac was observed, while a tendency to increased H3K4me1 was seen (Supplementary Fig. S3d). Demethylation of H3K4me3 and deacetylation of H3K27ac, particularly at CGI promoters, have been suggested to correlate with an increase of H3K27me3 (Refs^11,16,54^). However, we observed very little effects on H3K27me3 signals (Fig. 3g). At the first time points, no changes in H3K27me3 were detected and only after 16 h and beyond a few sites showed enhanced signals (Fig. 3g, Supplementary Table S6 and S7; available in GEO under accession number GSE240987). Despite the small numbers, the majority of changes observed occurred at promoters (Fig. 3h). These effects are also documented with integrative genomics viewer (IGV) browser snapshots comparing the different histone marks at the *Egr1* promoter (Supplementary Fig. S3e). The decrease in H3K4me3 in the promoter region was accompanied by an increase in H3K4me1, a small decrease in H3K27ac, and an increase in H3K27me3 at late time points. Despite the increase in mRNA after 30 min of dTAG-13 treatment, no obvious changes in histone modifications were apparent at this early time point (Fig. 2d and Supplementary Fig. S3e). The signals for ASH2L were weak and decreased within 1 h of dTAG-13 treatment (Supplementary Fig. S3e). Together, these findings support the conclusions of ordered changes in the studied histone marks as indicated by the genome-wide analyses.

Because the majority of sites with H3K4me3 loss, H3K4me1 gain, and H3K27ac loss are located within a window of ± 1 kb of TSSs, we evaluated whether these changes occurred at the same promoters. The large majority of H3K4me1 gains and H3K27ac losses overlapped with promoters that showed a decrease in H3K4me3 (Fig. 3i). This suggested that during the loss of H3K4me3, the H3K4me1 signals increased at least transiently at more than half of the promoters. This together with the decrease of H3K27ac might contribute to gene repression as suggested earlier^55,56^.

**Figure 4:**
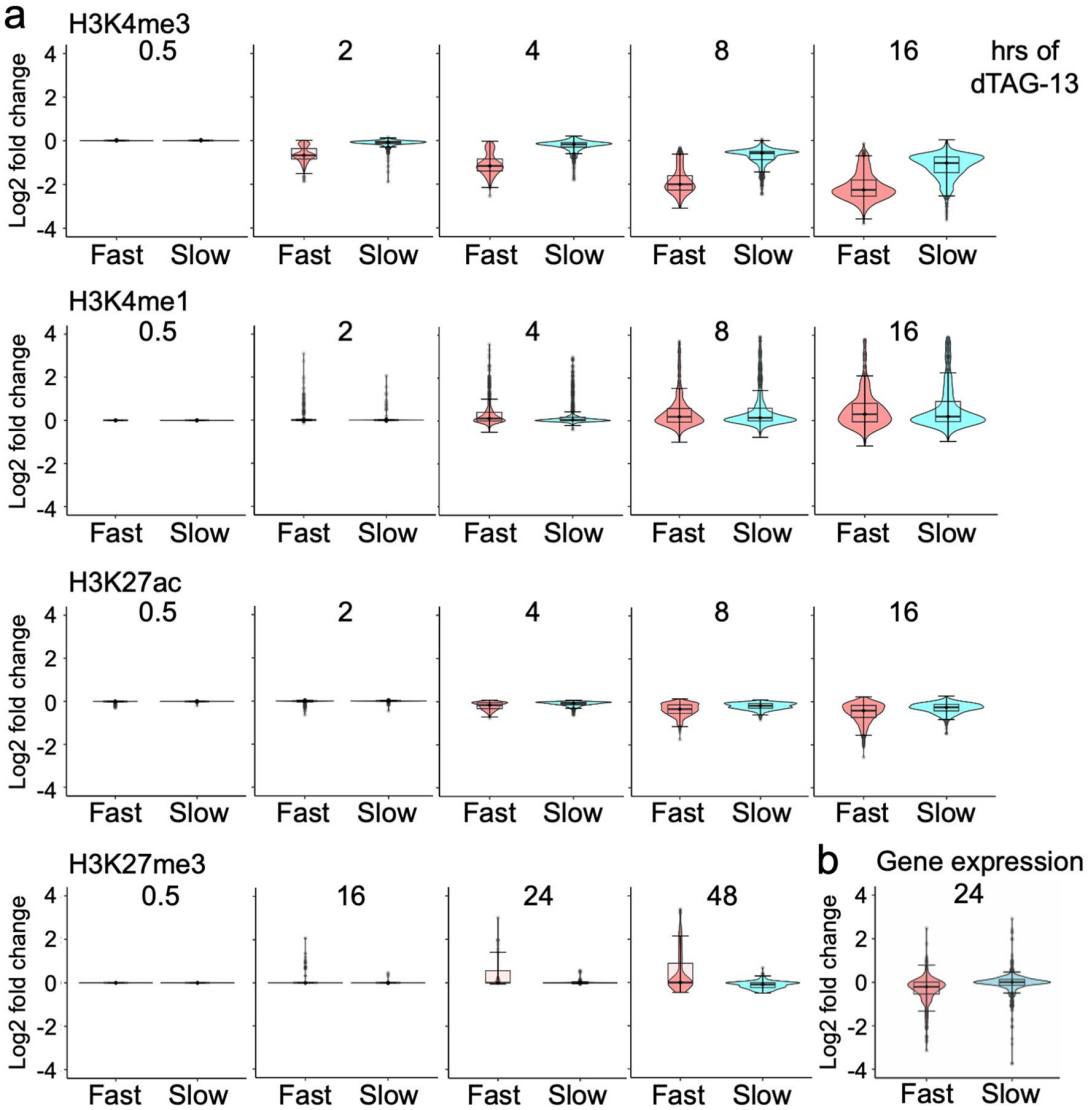
Promoters with a fast loss of H3K4me3 show more rapid alterations of other histone marks. (**a**) Two classes of promoters were chosen. Promoters that showed significant decrease in H3K4me3 signals either fast (499 at 2 h) or slow (788 at 16 h, excluding promoters that lost H3K4me3 signals after 30 min, 2 h, 4 h or 8 h) (q < 0.05; logFC > 0.58). A window of ± 1 kb around transcriptional start sites was considered. The log2 fold changes of signals of the indicated histone marks at the promoters of these two classes are displayed. For H3K27me3 the 2, 4 and 8 h time points are not shown as no changes were observed. (**b**) Gene expression as determined by RNA-seq after 24 h of dTAG-13 treatment was linked to promoters that lost H3K4me3 signals either fast or slow (as defined in panel **a**).

Although the effects on H3K27ac were small, the ChIP-qPCR analysis of selected genes suggested that the decrease in this histone mark can be rapid (Supplementary Fig. S3d). Therefore, we compared the histone marks of promoters that lost H3K4me3 signals either fast, i.e. during the first 2 h, or slow, i.e. only a significant loss at the 16 h time point (499 and 788 promoters, respectively; Fig. 4a). H3K4me1 and H3K27ac increased and decreased more rapidly, respectively, in the fast class. While the signals for H3K4me1 picked up at the later time points in the slow class, this was only minimal in the case of H3K27ac. For H3K27me3 an overall trend to increased signals at late time points was observed, particularly in the fast class, however, only few of these changes were statistically significant (compare to Fig. 3g,h). Furthermore, we divided the promoters with H3K4me3 signals into three equal groups, high, medium and low, according to H3K4me3 signal strength (Supplementary Fig. S4a), as applied before^46^. We noticed that the decrease over time was comparable. H3K4me1 and H3K27ac increased and decreased, respectively, faster in the high group. Interestingly, the trend to increased H3K27me3 was most prominent in the H3K4me3 high group. Thus, these comparisons suggest that different groups of promoters can be characterized by their varying degrees of H3K4me3 stability and distinct dynamics of other histone marks.

Together, these observations suggest an ordered sequence of events. The rapid loss of FKBP-HA_2_-ASH2L promotes first a decrease of H3K4me3 at promoters, followed by an increase in H3K4me1 and a decrease in H3K27ac, while changes in H3K27me3 appear late after FKBP-HA_2_-ASH2L depletion. To address whether this order of events affected gene expression, we evaluated the promoters that lost H3K4me3 fast or slow for the expression of the associated RNA. This revealed that the fast group, i.e. loss of H3K4me3 signals after 2 h, was preferentially associated with downregulated genes (Fig. 4b). The slow group, i.e. loss of H3K4me3 signals only after 16 h, no preference was observed for either up- or downregulated RNAs. We also asked whether the intensity of H3K4me3 at promoters might be a determinant for altered gene expression. However, the three equal groups of high, medium, and low H3K4me3 promoters did not stratify differentially regulated genes (Supplementary Fig. S4b). Thus, the timing of H3K4me3 loss rather than the level of this histone mark was relevant for gene expression.

### Changes in H3K4 and H3K27 marks occur with a slight preference at CpG island promoters

**Figure 5:**
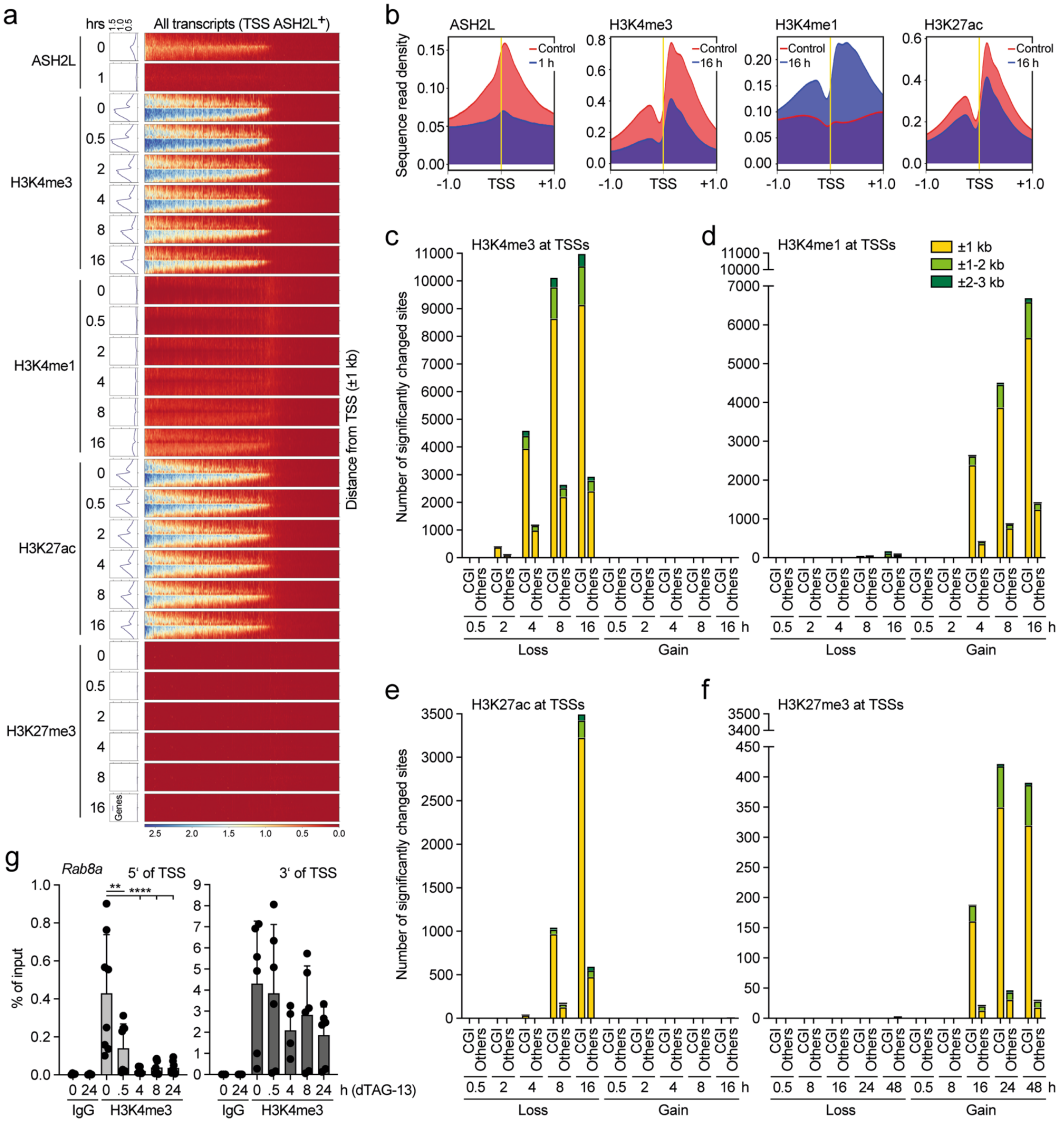
Decrease of H3K4me3 signals upon ASH2L loss is not prevalent at CpG island promoters. (**a**) Heatmaps and plot profiles generated using DeepTools showing the normalized ChIP-seq signals of the indicated histone marks in response to dTAG-13 treatment at ± 1 kb of TSSs of all annotated transcripts in mm10 that show an ASH2L signal in the promoter region of either of the two replicates (normalized using counts per Million). (**b**) Plot profiles generated using DeepTools show the average normalized signals of the indicated ChIP-seq data as in panel (**a**). (**c**–**f**) Lost and gained signals of H3K4me3 (panel **c**), H3K4me1 (panel **d**), H3K27ac (panel **e**), and H3K27me3 (panel **f**) were analyzed regarding their association with CpG island (CGI) promoters versus non-CGI promoters (CGIs were determined in the ± 1 kb window of TSSs). The signals relative to the TSS are summarized and the time points compared as indicated. (**g**) ChIP-qPCR experiments of H3K4me3 measured upstream (5′) or downstream (3′) of the TSS of *Rab8a*. Mean values ± SD of 4–8 experiments are shown. (** ≤ 0.01; ****p ≤ 0.0001).

Because the largest effects were observed at a ± 1 kb window centered around TSSs (Fig. 3), we compared all transcripts within the murine genome assembly mm10 with all four histone marks, i.e., H3K4me3, H3K4me1, H3K27ac and H3K27me3, and with FKBP-HA_2_-ASH2L signals. The ASH2L signals were centered close to TSSs that are positive for H3K4me3 (Fig. 5a,b). The biphasic pattern of H3K4me3 around the TSS with typically more signals 3′ of TSSs was not reflected in the FKBP-HA_2_-ASH2L binding pattern. Its binding was just slightly 3′ of the TSS (Fig. 5a,b). FKBP-HA_2_-ASH2L signals were strongly reduced within one hour of dTAG-13 treatment (Fig. 5a,b), consistent with the rapid decrease of the protein measured on Western blots (Fig. 1 and Supplementary Fig. S1). The signals for H3K4me3 and H3K27ac decreased, while H3K4me1 increased at promoters that are labeled with FKBP-HA_2_-ASH2L in control cells (Fig. 5a–e). Of note was that the distribution pattern of the gained H3K4me1 signals around TSSs was comparable to the pattern seen for H3K4me3, supporting the interpretation that H3K4me1 is the result of removing two methyl groups from H3K4me3 (Fig. 5b). The signals for H3K27me3 were very weak and, at the resolution used, no changes could be observed during the first 8 h. The number of signals remained low at later time points (Fig. 5a,f).

Our previous studies indicated that CGIs are particularly sensitive to altered KMT2 activities^46^, consistent with previous suggestions^11,16,54^. However, it was unclear whether this was a secondary effect of the slow loss of Ash2l upon recombination. It has been suggested that roughly 70% of vertebrate promoters are characterized by a CGI^57,58^. Of the 25,431 H3K4me3 peaks, 18,791 are promoter-associated. Of these 79% are characterized by a CGI. To assess whether changes in histone marks are preferentially at CGI promoters upon short-term regulation of FKBP-HA_2_-ASH2L, we compared the changes in the four histone marks H3K4me3, H3K4me1, H3K27ac and H3K27me3 regarding their association with CGI positive and negative promoters. As soon as significant changes in signal intensities were detected, the majority of these changes were associated with CGI promoters (Fig. 5c–f). Of the promoters with a decrease of H3K4me3 signal in the ± 3 kb window of TSSs, 79% were categorized as CGI promoters both after 8 and 16 h (Fig. 5c). For those that gained H3K4me1 signals, the proportion of CGI promoters was slightly higher, 84% and 82% after 8 and 16 h, respectively (Fig. 5d). Similar numbers were obtained for promoters that lost H3K27ac signals, 85% after 8 and 16 h (Fig. 5e). The preference for CGI promoters was higher when H3K27me3 gains were analyzed with 90% and 93% after 8 and 16 h, respectively, although the numbers are small and therefore the conclusion may not be robust (Fig. 5f). Thus, short-term regulation of FKBP-HA_2_-ASH2L did not reveal a preference for CGI promoters regarding H3K4me3. Nevertheless, the consequences, i.e. the measured increase in H3K4me1, decrease in H3K27ac, and increase in H3K27me3, were slightly higher for CGI promoters than predicted. In other words, promoters that do not possess a CGI seem slightly less responsive to the downstream consequences of FKBP-HA_2_-ASH2L and H3K4me3 loss, at least at these early time points. Also, similar to the findings upon *Ash2l* KO^46^, the fold decrease of H3K4me3 was higher upstream of the TSS as exemplified for the *Rab8a* promoter (Fig. 5b,g). Together, this suggests that CGI promoters are only slightly more sensitive to KMT2 complexes upon short-term FKBP-HA_2_-ASH2L loss, unlike what we observed previously^46^.

### Loss of ASH2L alters chromatin accessibility

**Figure 6:**
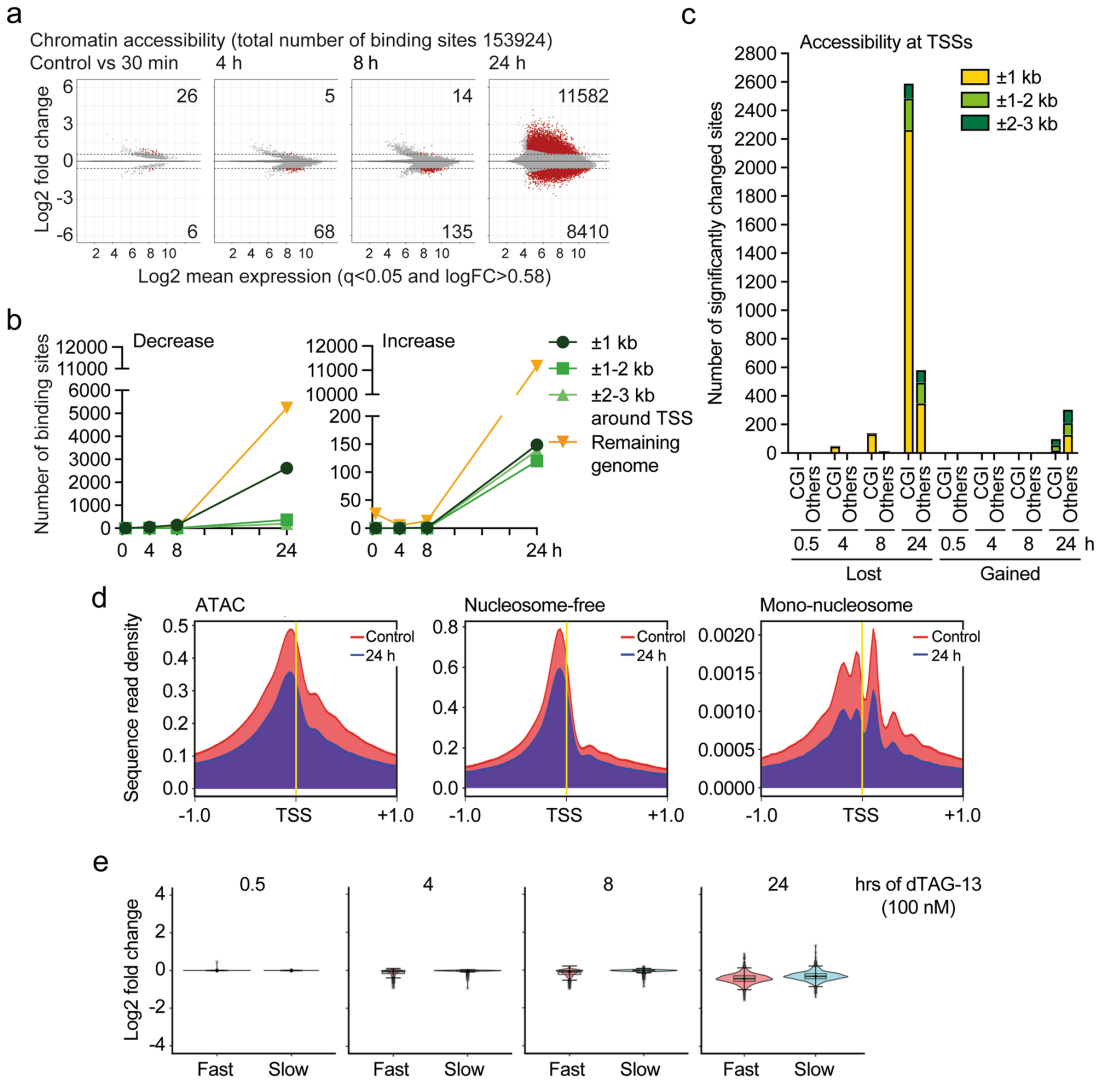
Loss of ASH2L reduces accessibility and decreases regularity at promoters. (**a**) ATAC-seq analyses were performed on NG3 cells treated with or without dTAG-13 (100 nM) for the indicated times. Displayed are MA plots. Given are the total number of sites identified across all samples and the sites that were significantly more or less accessible (red dots: q < 0.05 and logFC > 0.58) upon loss of FKBP-HA_2_-ASH2L. (**b**) Summary graphs that detail the annotation of significantly changed sites (q < 0.05 and logFC > 0.58) associated with transcriptional start sites (TSSs) compared to the remaining genome. (**c**) Promoter-associated ATAC-seq sites with significantly increased or decreased signal intensity were assigned to promoters with or without CpG islands (CGIs were determined in the ± 1 kb window of TSSs). (**d**) Plot profiles generated using DeepTools showing the average normalized signals of the ATAC-seq data are plotted ± 1 kb of TSSs of all annotated transcripts in mm10 (normalized using counts per Million). Nucleosome-free and mono-nucleosomes refer to ATAC-seq fragments that are smaller than 120 bp and between 130 and 200 bp, respectively. (**e**) Promoters with significant decrease in H3K4me3 signals within 2 h (499, fast) or after 16 h (788, slow, excluding promoters that lost H3K4me3 signals after 30 min, 2, 4 or 8 h) (q < 0.05; logFC > 0.58). A window of ± 1 kb around TSSs was considered. The log2 fold changes of the ATAC-seq signals at the promoters of these two classes are displayed.

The loss of H3K4me3 at promoters and the decrease in gene expression of many genes suggested that promoters may become less accessible. ATAC-seq experiments revealed changes in accessibility that reached 13% of the total accessible sites (153,924) across all samples (Fig. 6a, Supplementary Table S8a and b; available in GEO under accession number GSE240987). The number of sites with increased and decreased accessibility was roughly equal. Of these, a proportion was associated with the ± 1 kb window surrounding TSSs, but most of the changes were observed in regions that were not associated with promoters, both for regions that gained and lost signals (Fig. 6b). For promoter-associated sites, a slight preference for CGI promoters was observed comprising roughly 82% after 24 h (Fig. 6c), and thus being comparable to the observations made for H3K4me3, H3K4me1 and H3K27ac (Fig. 5). Although the numbers were small, CGI promoters with gained ATAC signals were underrepresented with 25% (Fig. 6c). Overall, the accessibility at promoters decreased over time (Fig. 6b–d and Supplementary Fig. S5a–c), exemplified at the *Egr1* locus (Supplementary Fig. S3e). The nucleosome-depleted regions, ATAC fragments that are smaller than 120 bp, and well-positioned nucleosomes, ATAC fragments between 130 and 200 bp, at promoters decreased (Fig. 6d and Supplementary Fig. S5b,c). This is consistent with an increase in histone H3 binding at selected promoters (Supplementary Fig. S5d,e), similar to our previous observations in the *Ash2l* KO system^46^. Together, these findings support the notion that chromatin compaction increased. One expectation was that the precise positioning of a nucleosome upstream of the TSS would increase. However, both the nucleosome-free and mono-nucleosome signals decreased (Fig. 6d), suggesting that the increased protection is not due to precise positioning of nucleosomes. Rather, chromatin seems to appear less ordered. These changes were slow with little difference when the promoters of the fast and slow classes were compared (Fig. 6e) or the groups with high, medium and low H3K4me3 (Supplementary Fig. S5f). This suggests that they are not a direct consequence of ASH2L loss but that they occur downstream of altered histone modifications, including loss of H3K4me3, gain of H3K4me1 and loss of H3K27ac.

### Reduced chromatin accessibility at enhancers

**Figure 7:**
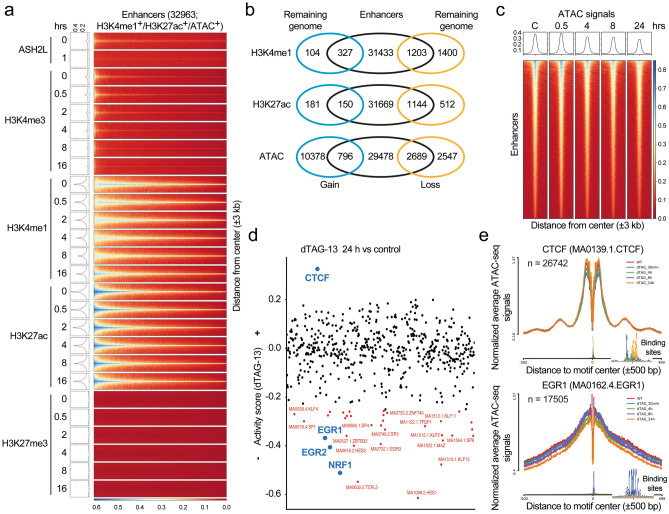
Minor alterations at enhancer upon loss of ASH2L. (**a**) Enhancers were defined by positivity for H3K4me1, H3K27ac and ATAC signals excluding signals that overlap with ± 1 kb of transcriptional start sites (TSSs) of the known mm10 promoters. Heatmaps and plot profiles generated using DeepTools show the indicated ChIP-seq signals (normalized using counts per Million) of Ash2l and the indicated histone marks in response to dTAG-13 treatment. The signals at ± 3 kb of the center of the 32,963 identified enhancers are displayed. (**b**) Summarized are the overlaps between significantly changed H3K4me1, H3K27ac, and ATAC sites (q < 0.05 and logFC > 0.58; the 16 and 24 h time points for H3K4me1 and H3K27ac and for ATAC-seq, respectively) that are intergenic (more than 3 kb from TSSs of all transcripts in mm10) and the list of enhancers identified as in panel a. Remaining genome refers to the intergenic sites that are not showing overlap with the enhancers as defined here. (**c**) Heatmaps and plot profiles as defined in panel (**a**) for ATAC-seq signals centered at enhancers (± 3 kb). (**d**) Analysis of transcription factor (TF) footprinting and their differential activity within the ATAC-seq dataset were conducted using the RGT-HINT tool. TFs highlighted in red are those that have over 1000 identified binding sites and showed significant change (q < 0.05) upon addition of dTAG-13 at 24 h. (**e**) Examples of line plots for CTCF (enhanced) and EGR1 (reduced) upon loss of FKBP-HA_2_-ASH2L. The inserts show the consensus sequence. The indicated numbers (n) are the identified binding sites for the respective TF.

To define enhancers, we combined the ATAC, H3K4me1 and H3K27ac signals that were not associated with promoters, i.e., excluding signals associated with a window of ± 1 kb of TSSs. This analysis resulted in 32,963 putative enhancers regions (Fig. 7a–c, Supplementary Table S9; available in GEO under accession number GSE240987). These regions were also positive for FKBP-HA_2_-ASH2L, which was broadly reduced upon dTAG-13 treatment (Fig. 7a). Moreover, a substantial fraction of the lost H3K4me1 and H3K27ac signals were associated with enhancers at the 16 h time point, while only few signal gains were measured (Fig. 7b). The majority of altered ATAC-seq signals (24 h time point) associated with the putative enhancer fraction were reduced, unlike the sites in the remaining genome that increased (Fig. 7b,c). It is important to note that the majority of the enhancers defined by ATAC, H3K4me1 and H3K27ac signals were not affected upon loss of FKBP-HA_2_-ASH2L when ATAC, H3K4me1 and H3K27ac signals were analyzed. The relatively little changes in H3K4me1 suggests that demethylation at enhancers is slow, similar to the observation at promoters. The H3K4me3 signals were low at enhancers and further decreased over time, while the H3K27me3 signals were very low (Fig. 7a). These findings suggest that the two characteristic histone marks at enhancers, H3K4me1 and H3K27ac, were predominantly downregulated upon loss of FKBP-HA_2_-ASH2L and that accessibility decreased.

The altered accessibility of chromatin, both at promoters and enhancers, suggested that binding sites of transcription factors might be affected. Therefore, the ATAC fragments were screened for transcription factor (TF) binding sites. Because little effects were observed at early time points (Fig. 6a), we focused on the treatment with dTAG-13 for 24 h. We considered sites that significantly changed upon loss of FKBP-HA_2_-ASH2L (*p* < 0.05) and that were represented by at least 1000 binding sites (Supplementary Table S10; available in GEO under accession number GSE240987). With these criteria, more than 30 motifs showed reduced accessibility, including those for EGR1, EGR2 and NRF1 (Fig. 7d). In contrast, only CTCF consensus binding sites showed enhanced binding activity, as previously observed in the KO system^46^. The alterations were time-dependent, exemplified for CTCF and Egr1, but rather small at early time points (Fig. 7e). Thus, the changes in histone marks and chromatin accessibility reduced occupancy of many transcription factor binding sites, compatible with overall decreased accessibility and altered gene transcription.

### Transcriptional rate upon loss of ASH2L

**Figure 8:**
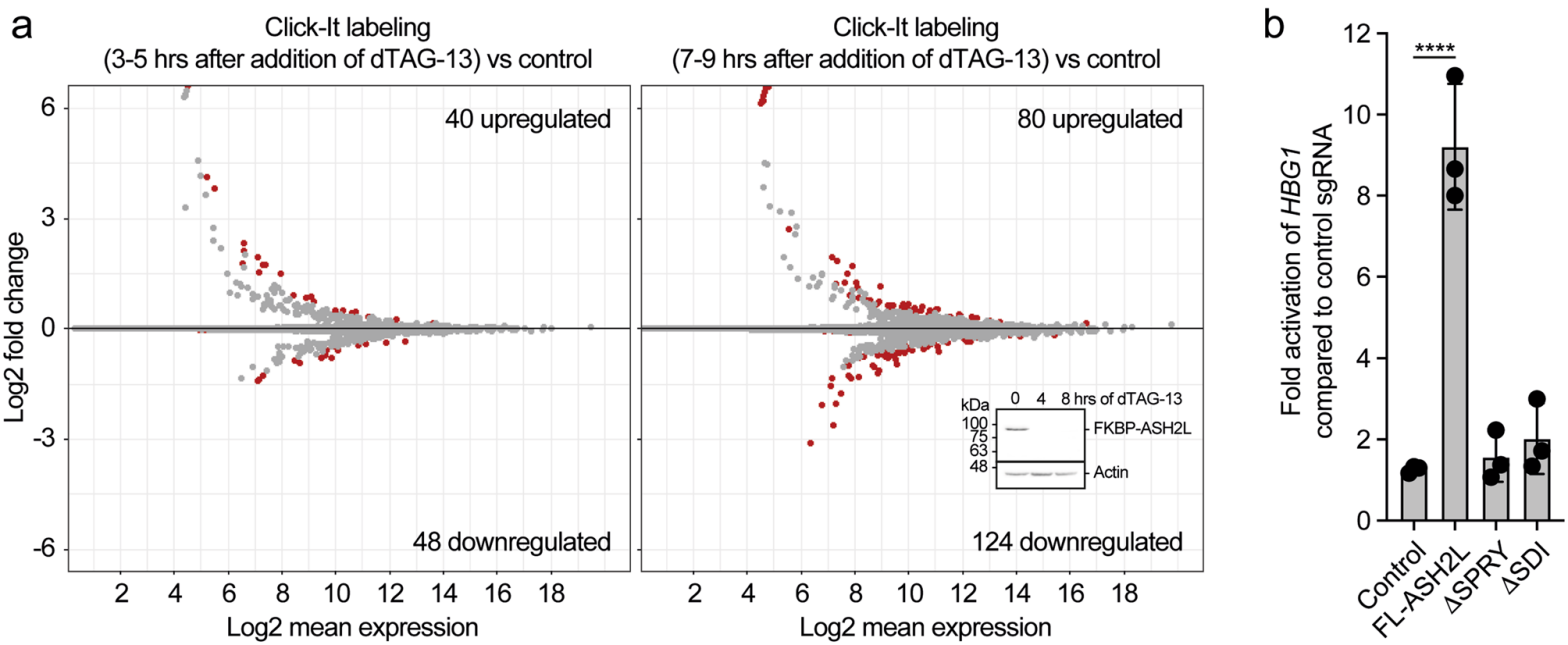
Promoter recruitment of ASH2L stimulates gene expression. (**a**) Cells were labeled with EdU after the addition of dTAG-13 as indicated. The modified RNA was isolated and analyzed by 3′ end sequencing. MA plots are displayed. Given are the total number of significantly upregulated and downregulated transcripts (red dots; q < 0.05). The data summarize two replicates normalized using ERCC spike-in RNA. The insert shows FKBP-HA_2_-ASH2L protein expression in control cells and cells treated with dTAG-13 for the indicated times. (**b**) Plasmids expressing dCAS9 and 3 different sgRNAs containing MS2 binding loops were transfected into HEK293 cells. The sgRNAs target the *HBG1* locus. For control, sgRNAs with no specificity for *HBG1* were used. RT-qPCR analysis of *HBG1* RNA was measured, compared to *GUS* RNA and ratios between the measurements of specific sgRNAs vs control sgRNAs are displayed. Mean values ± SD of three experiments. (****p ≤ 0.0001).

The loss of positive histone marks as well as reduced accessibility at both promoters and enhancers suggested that this affects gene transcription. Indeed, many genes were deregulated in response to FKBP-HA_2_-ASH2L loss after 24 h (Fig. 2). To address whether these observations resulted in altered transcription at early time points, we measured newly synthesized RNA by incubating the cells with 5-ethynyl uridine from 3 to 5 and 7 to 9 h after addition of dTAG-13. The modified RNAs were biotinylated (Click-It), poly-A selected, and sequenced. For control, the expression of FKBP-HA_2_-ASH2L was visualized in parallel samples (Fig. 8a insert). The analysis revealed that few genes were altered (Fig. 8a, Supplementary Table S11; available in GEO under accession number GSE240987). Thus, at these early time points the loss of FKBP-HA_2_-ASH2L and the decrease in H3K4me3 at promoters did not have an immediate consequence on gene transcription. We conclude that the loss of FKBP-HA_2_-ASH2L per se was not sufficient to alter gene expression. It is likely that at later time points effects will become apparent, with the drawback that secondary effects may influence the outcome. We determined whether the small number of deregulated transcripts were associated with genes whose promoters lost H3K4me3 fast or slow (Supplementary Fig. S6a). Also we asked whether the level of H3K4me3 at promoters was linked to altered transcription (Supplementary Fig. S6b). These analyses did not reveal any clear association.

To address whether ASH2L can activate gene transcription, we employed a dCAS9 system with sgRNAs containing MS2 binding sites targeting the *HBG1* gene locus^59,60^. In HEK293 cells MS2-ASH2L was sufficient to activate the expression of the chromatin-embedded *HBG1* locus (Fig. 8b). ASH2L interacts with the KMT2 complex core components RBBP5 and DPY30 through the SPRY and the SDI domains, respectively^27,61^. These interactions are necessary for efficient methyltransferase activity and for positioning the complexes to specific sites in chromatin. Mutants in ASH2L that do not interact with RBBP5 and DPY30, MS2-ASH2LΔSPRY and MS2-ASH2LΔSDI, respectively, were unable to induce gene expression (Fig. 8b). Thus, positioning ASH2L, dependent on its ability to interact with its direct partners, is sufficient to induce gene expression, supporting the conclusion that H3K4me3 contributes to transcription^62^.

## Discussion

PROTAC systems have been established for all four WRAD core complex components, including our study^62–64^. This has allowed studying COMPASS/KMT2 complexes and their activities in unprecedented detail. This is important because of the central and broad activities of these complexes. This includes and is best studied for the modification of both promoters and enhancers with H3K4me3 and H3K4me1, respectively. These histone marks are closely linked to gene expression. Obviously, loss of KMT2 complexes and as a consequence altered modification of H3K4 methylation results in very broad effects on gene transcription and RNA expression. Some of these effects are direct but others, probably the majority, are indirect and thus the consequences on biological processes are extensive. The rapid degradation of WDR5, RBBP5, ASH2L and DPY30, in all cases the proteins were lost very rapidly (Figs. 1 and S1; Refs^62–64^), allows now to address the direct consequences more specifically. A common consequence is a decrease of H3K4me3 and less prominently H3K4me1, and broadly altered gene transcription. Because the stability of the H3K4 methylation is different when tri-methylation is compared to mono-methylation, the demethylases for these modifications appear to have distinct activities and possibly also distinct mechanisms how they are targeted to chromatin sites. Both knockout and inhibitor studies support an important role for KDM5 family members^62,64^, which are thought to be major H3K4me3 and me2 demethylases^8^. Thus, the dynamics of H3K4 methylation/demethylation is complex and most likely promoter specific.

The dynamics upon loss of WRAD components seem to be different between experimental systems. For example, the effects on H3K4 methylation were much faster in one system of mESCs^62^, but considerably slower in another^64^, the latter being comparable to our iMEF cells. A major difference between mESCs employed in Wang et al. (Ref^62^) and our iMEF cells is that the former grow considerably faster, roughly 12 vs 24 h doubling time. This rapid cell cycle progression may require that adjustments to H3K4me3 levels at promoters are much faster with more rapid turnover rates. Indeed, both global as well as promoter-associated H3K4me3 is more stable in our cells (Figs. 1, 2) than in the mESCs^62^. This is also consistent with the changes in gene transcription that are much faster in mESCs compared to the iMEF cells, despite the three WRAD components ASH2L, DPY30, and RBBP5 being equally fast degraded upon addition of a PROTAC. In the slower mESC system^64^, the effects have a comparable dynamic as observed in our iMEF cells. Together, these findings provide an explanation as to why the timing of downstream effects may not be easily comparable (see also below).

ASH2L is necessary for proliferation, a conclusion that is supported by the observations in the PROTAC system shown here (Fig. 1), and by our previous findings upon knockout of *Ash2l* in MEF cells^47^. Moreover, the knockout of *Ash2l* both in the liver and in the hematopoietic system inhibits proliferation^29,65^. Similarly, the knockout of *Dpy30* prevents proliferation of hematopoietic cells^42^. The knockout of *Wdr5* in mouse embryonic stem cells (mESCs) blocks the ability of these cells to form colonies^66^. Knockdown studies in tumor cells revealed that the different WRAD subunits are important for proliferation to various degrees^10^. In mESCs, which proliferate very fast, the efficient knockdown using PROTAC systems for Rbbp5 and Dpy30 increased duplication time from about 12 to 24 h^62^. Thus, both the Rbbp5 and the Dpy30 knockdown cells still proliferate exponentially at least until day 6. Interestingly, the colony formation assays were more decisive, possibly because this is more stressful for cells^62^. Thus, all WRAD subunits are necessary for proliferation to various degrees in the different cell types that have been analyzed. Moreover, the heterogenous effects observed at enhancer and promoters and when RNA is analyzed support the observation that upon loss of ASH2L cells arrest throughout the cell cycle. We interpret this observation to the effect that many different key factors responsible for processes such as cell cycle progression and replication may become limiting in individual cells. As a result, cells arrest in different phases of the cell cycle and are unable to continue into the next cell cycle phase in the absence of ASH2L. We suggest that this most likely reflects the reduced capacity to activate gene transcription. This may prevent the accumulation of cells in one defined cell cycle phase such as G0/G1. Single cell analyses will be required to address this in more detail.

The pattern of H3K4me3 in control cells and of H3K4me1 in cells after loss of FKBP-HA_2_-ASH2L is similar around TSSs (Figs. 3, 5). The 5′ signals are typically weaker than the 3′ signals, while the immediate vicinity of the TSS shows little signal. This latter region is particularly accessible in the ATAC experiments and is the region that binds ASH2L (Fig. 5). Thus, it appears that KMT2 complexes interact with the nucleosome-free region, possibly by binding to the RNAPII through WDR82^67–69^, or recruitment of KMT2 complexes by other means, including sequence-specific transcription factors, that connect to the RNAPII complex bound at the TSS^27^. This region loses accessibility upon FKBP-HA_2_-ASH2L loss and nucleosomes are less well-positioned (Fig. 6 and Supplementary Fig. S5). Thus, loss of accessibility is not correlated with a stabilization of a well-positioned mono-nucleosome upstream of TSSs. In this respect it is of note that Set1A and B are located mainly downstream of the TSS^69^. Of note, the pattern of Set1A and B binding closely reflects the distribution of H3K4me3 at promoters, while the ASH2L signals are not biphasic (Fig. 5). It will be interesting to see whether other KMT2 enzymes are similarly positioned.

The lack of altered H3K27me3 at promoters was somewhat unexpected as H3K4me3 has been suggested to interfere with Polycomb repressive complex 2 (PRC2), the complex that methylates H3K27^70^. This interference can be due to competing interactions of COMPASS and PRC2 with histone tails and/or by direct inhibition of PRC2 by H3K4me3^71–73^. Whether the remaining H3K4me3 and/or H3K27ac marks are sufficient to prevent H3K27me3 remains open. It is possible that relevant thresholds of these modifications have not been reached for efficient PRC2 recruitment. Alternatively, loss of KMT2 complexes and the decrease of H3K4me3 is not sufficient to promote PRC2 recruitment and activity, for example, because positive signals are lacking. PRC2 recruitment is complex, a major determinant being a high CpG content as in CGIs, but other mechanisms have also been discussed^70,74^. Further studies need to address more directly whether loss of ASH2L alters the binding of PRC2 complexes.

The recent findings suggest that RNAPII is affected by H3K4 methylation and KMT2 complexes. An important early suggestion was that H3K4me3 helps recruit RNAPII complexes as the TFIID subcomplex can directly read this histone mark^75,76^. However, in the PROTAC-mediated loss of RBBP5 and DPY30, no effect on TFIID recruitment was observed^64^. Additional mechanisms have been described, including binding by sequence-specific transcription factors and binding to chromatin^27^. Moreover, interactions with CGI promoters have been documented^11^, which is particularly relevant for SET1A and B^69^. Together, these findings provide further evidence of the complexity of the system, with multiple mechanisms being relevant for the interplay of COMPASS with RNAPII complexes. The recent findings suggest that direct recruitment of RNAPII complexes seems independent of the presence of COMPASS/KMT2 complexes^62,64,69^, consistent with the observation that transcription rate is not affected early after loss of ASH2L (Fig. 8). This is also in agreement with RNAPII loading being unaffected upon loss of RBBP5 early but not late after activation of protein degradation^64^. Nevertheless, the role of H3K4me3 is still not fully understood as even small amounts of this histone mark may be sufficient to control RNAPII loading. Unlike loading, COMPASS complexes are suggested to regulate pausing of RNAPII and premature termination, although the findings are not fully consistent at present^62,64,69^.

The recent findings discussed above strengthen the correlation between recruitment of KMT2 complexes to promoters, the subsequent tri-methylation of H3K4, and as a consequence effects on gene transcription. This is further supported by positioning the ASH2L protein through dCAS9 to a specific, endogenous promoter. In this setting, ASH2L is sufficient to activate expression of *HBG1* (Fig. 8). This requires the SPRY domain, which interacts with RBBP5, and the SDI domain, which binds DPY30. Thus, it appears that at least the other WRAD complex subunits, but most likely also one of the KMT2 catalytic subunits, need to be present for activating gene expression.

In summary, our study on the PROTAC-dependent, rapid degradation of ASH2L, provides evidence for a hierarchical alteration of histone marks. While reduction of H3K4me3 occurs first, gain and loss of H3K4me1 at promoters and enhancers, respectively, follows, succeeded by a decrease of H3K27ac at both promoters and enhancers. Similarly, a reduction in chromatin accessibility is occurring late, resulting in less structured nucleosomal organization at promoters, while H3K27me3 is only minimally affected. The early loss of H3K4me3 is associated with gene repression, while the level of H3K4me3 at promoters appears not to be a determinant that influences gene expression. Thus, also the initial event, the degradation of ASH2L is fast, subsequent chromatin-associated steps as well as regulation of gene expression are rather slow, which argues for a strong buffering effect built into the system. This might be due to additional histone marks, which are manifold and most of them only poorly understood mechanistically^5,77,78^. Also, thresholds of distinct histone modifications might influence RNAPII loading and activity. Moreover, it is well possible that H3K4 methylation has a facilitating function, which in the absence of activating or repressing signals results in small effects on gene transcription. However, these will be amplified in feedback loops once critical components for transcription become limiting or unbalanced. Subsequently, this will lead to broad deregulation of many genes, compromising many different cellular processes as seen in our study. Together, PROTOAC systems as used here and published by others while our work was in progress^62,64,69^, support a role of H3K4 methylation in controlling gene transcription and will allow further detailed evaluation to define functions of COMPASS/KMT2 complexes.

## Material and methods

### Protein analyses

SDS-PAGE and Western blotting have been performed identically to the procedure described previously and the antibodies used have been listed^47^. Full size blots are shown in Supplementary Figs. S7 and S8. The antibodies used are summarized in Table 1.

**Table 1:** Summary of the antibodies used.

Antigen	Origin	Clonality	Co./Cat.No	RRID number	Purpose
*Primary antibodies*					
Actin	Mouse	MC-C4	MP Biochemicals #691001	AB_2335304	WB
Ash2l	Rabbit	MC-D93F6	Cell Signaling #5019	AB_1950350	WB/IF
γ-H2A.X	Mouse	MC	Millipore 05-636	AB_309864	WB
H3	Rabbit	PC	Abcam #ab1791	AB_302613	WB/ChIP
H3K4me1	Rabbit	PC	Abcam #ab8895	AB_306847	WB/ChIP
H3K4me3	Rabbit	PC	Abcam #ab8580	AB_306649	WB/ChIP
H3K27ac	Rabbit	PC	Abcam #ab4729	AB_2118291	WB/ChIP
H3K27me3	Rabbit	PC	Antibodies Online #ABIN6923144	–	WB/ChIP
H3K27me3	Rabbit	PC	Antibodies Online #ABIN6952339	–	WB/ChIP
HA	Mouse	MC-B1612	Covance/Biolegend 901513	AB_2565336	WB/IF
HA	Rabbit	MC-C29F4	Cell signaling #3724S	AB_1549585	ChIP
IgG	Rabbit	PC	Diagenode #C01010080	AB_2722553	ChIP
Rbbp5	Rabbit	PC	Bethyl #A300-109A	AB_210551	WB
p53	Mouse	MC-1C12	Cell signaling #2524	AB_331743	WB
γ-Tubulin	Mouse	MC-GTU88	Sigma-Aldrich #T5236	AB_532292	WB
Wdr5	Rabbit	PC	Bethyl #A302-429A	AB_1944302	WB
Wdr5	Rabbit	PC	Abcam # ab22512	AB_2215559	WB
*Secondary antibodies*					
Mouse IgG-HRP	Rat	PC	Jackson Immuno Res., 415-035-166	AB_2340269	WB
Rabbit IgG-HRP	Goat	PC	Jackson Immuno Res., 111-035-144	AB_2307391	WB
Mouse IgG AF488	Donkey	PC	Invitrogen #A21202	AB_141607	IF
Rabbit IgG AF488	Goat	PC	MolecularProbes #A11008	AB_143165	IF

### RNA isolation and cDNA synthesis

RNA purification from cells was performed using the HighPure RNA Isolation Kit (Roche, 11826665001). The RNA was transcribed into cDNA by using the QuantiTect Reverse Transcription Kit (Qiagen, 205314). For *Il7* and *Egr1* QuantiTect primers (QT00101318 and QT00265846, respectively, Qiagen) were used for quantitative PCR. Primers for *Nrf1* were: forward, GAGAATGTGGTGCGAAAGT; reverse, GCTCTGAATTAACCTCCTGTG; for *Ppbp*: forward, ACCATCTCTGGAATCCCATTCA; reverse, GTCCATTCTTCAGTGTGGCTATC.

### Cloning and vector design

The human ASH2L sequence was introduced to the lentiviral vector pLEX305-NdTAG (Addgene#91797) using a Gateway LR reaction (Invitrogen). The LR reactions were performed overnight at 25 °C in a final volume of 10 μl according to the manufacturer’s instruction and used to transform competent Stbl3 bacteria. After plasmid preparation the integrity of the Gateway expression constructs was controlled by restriction digest.

### Cell culture and lentiviral transduction

HEK293T and immortalized mouse embryonic fibroblast (iMEF^47^) cells were cultured in DMEM supplemented with 10% (v/v) FBS and 1% P/S (v/v) at 37 °C in a humidified incubator at 5% CO2.

For lentiviral vector production, a third-generation packaging system was transfected into HEK293T cells, including three helper plasmids (pMDLg/pRRE, pRSV-Rev, pCMV-VSV-G), pLEX-305-N-dTAG-hASH2L expressing FKBP-HA_2_-ASH2L, and pH2B-YFP to estimate transfection efficiency. After 48 h, the cell culture supernatant was collected and passed through a 0.45 µm PVDF filter. iMEF cells were seeded at a confluency of ~ 40% and incubated with lentivirus-containing supernatant, diluted 1:2 with fresh medium, on the next day. Polybrene (8 µg/mL) was included in the infection protocol. The medium was changed after 6–8 h and puromycin (8 µg/mL) was added the following day. In order to eliminate the endogenous *Ash2l*, transduced cells were treated with 5 nM of 4-hydroxytamoxifen for 3 days to excise exon 4 of the endogenous *Ash2l* loci. Individual clones were then established using limiting dilution and expanded.

### Cell proliferation assay

For cell proliferation assays, three biological replicates, each in triplicates, were performed. Five × 10^4^ cells were seeded in a 6-well plate and dTAG-13 treatment (100 nM) immediately. Cells were counted at days as indicated in the figure. Before cells reached confluency, they were split at a ratio of 1:2–1:3.

### Cell cycle analysis

Cells were harvested by trypsinization, collected by centrifugation and washed in ice-cold phosphate-buffered saline (PBS). The cells were then fixed using 4% paraformaldehyde in PBS, and stained with Vybrant™ DyeCycle™ Violet Stain (Invitrogen™, #V35003) at the final concentration of 5 µM at 37 °C for 30 min. Violet signal was acquired in the Pacific Blue channel in a linear mode with low speed. The percentage of the cell population in distinct phases of the cell cycle was determined using a manual gating method. G1 gate width was considered the same as G2 gate width (unconstrained), and the area under the curve was calculated as cell percentage.

### EdU incorporation assay

Click-iT™ EdU Alexa Fluor™ 488 (AF488) Flow Cytometry Assay kit (Invitrogen) was used to analyze DNA synthesis essentially according to the manufacturer’s instruction. The labeling with EdU (5-ethynyl-2′-deoxyuridine) was done at a final concentration of 10 µM for 3 h prior to harvesting. The DNA was stained with Vybrant DyeCycle Violet. The permeabilized cells were incubated with AF488 azide to modify the incorporated EdU. Violet and AF488 signals were acquired in the Pacific Blue (Lin), and FITC (Log) channels, respectively. The percentage of FITC-positive cells was determined using manual gating with a threshold set above the FITC signal from non-EdU treated samples.

### RNA-Seq

RNA was isolated as described above. For quality control, samples were analyzed using an RNA ScreenTape (Agilent, 5067-5576) with a TapeStation-device (Agilent). For internal control, ERCC-RNA-spike-in (ThermoFisher, 4456740) was added to every sample. For library generation the Collibri 3′-mRNA Prep Kit (ThermoFisher, A38110024) was used. Sequencing was performed on a NextSeq500/550 platform (Illumina) based on a Mid Output Kit 2.5 (Illumina, 20224904) using 75 cycles and single-end reads. Quality control, library preparation and sequencing were executed by the Genomics Core Facility of the Interdisciplinary Center for Clinical Research (IZKF) of the Medicine Faculty at RWTH Aachen University.

### Chromatin-immunoprecipitation (ChIP)

ChIP experiments were carried out using the ChIP-IT High Sensitivity^®^ kit (ActiveMotif) essentially according to the manufacturer’s instruction. Modifications were as follows. For nuclei isolation, 60–70 strokes were applied using a 5 mL glass dounce homogenizer with a tight (B) pestle. The amount of chromatin used per IP was 30 µg and 100 µg for histone marks and ASH2L, respectively. Chromatin shearing was conducted using the Bioruptor^®^ Pico sonication device. Sonication was performed in 300 µL aliquots in 1.5 mL Bioruptor^®^ Pico microtube for 4–5 rounds of 10 cycles (each cycle was 30 s sonication/30 s pause) until the majority of chromatin fragments were sheared down to ~ 200 and to ~ 500 bp (for ChIP-seq and ChIP-RT-qPCR, respectively). Input DNA was precipitated by adding 2 µL of carrier (provided by the kit) and 2 µL glycogen (20 mg/mL). For sequencing, the concentration of samples was measured using the Quantus™ Fluorometer. Sample quality control/fragment size distribution was assessed using the Bioanalyzer system (Agilent). Samples were then indexed and adaptor-ligated using NEBNEXT Ultra II DNA Library Preparation Kit (NEB) according to the manufacturer’s instructions. For all histone marks (except H3K27me3, known as broad histone mark) a ~ 350 bp (200 bp chromatin fragment size + 120 bp two adaptors size) size selection step was conducted prior to PCR library amplification. Sequencing was performed on a NextSeq 550 (Illumina) system using a NextSeq 500/550 High Output v2.5 (75 Cycles) cartridge (Single-End). The number of samples per cartridge was arranged in a way to provide a minimum of 40–50 M raw reads per ChIP sample.

### ATAC seq

Chromatin accessibility was performed as described^46,79–81^. In brief, cells were treated with dTAG-13 or EtOH for control, collected and washed in PBS. Subsequently, ATAC lysis buffer (10 mM Tris–HCl, pH 7.4, 10 mM NaCl, 3 mM MgCl_2_, 0.1% NP-40, 0.1% Tween-20, 0.01% Digitonin) was added. Nuclei were pelleted and incubated in transposition mix (1× TD (Illumina, 20034210), 7.5 μl per sample TDE1 (Illumina, 20034210), 0.1% Tween-20, 0.01% Digitonin, in PBS) in a final volume of 50 μl per sample at 37 °C for 1 h. Tagmented fragments were purified using the MinElute PCR purification kit (Qiagen, 28004). The transposed DNA fragments were amplified using 25 µM Nextera i5 and i7 barcoded primers in NEB Next Ultra II Q5 Master Mix (New England Labs, M0544). DNA purification was performed using AMPureXP magnetic beads (Beckman Coulter, A63880) in 96-well plates. The beads were washed with 200 µl of 85% ethanol. The DNA was eluted in water and stored in DNA low-binding tubes.

### Click-It-seq

Newly synthesized transcripts were analyzed using the Click-iT™ Nascent RNA Capture Kit (Invitrogen). The cells were incubated with 0.2 mM EdU for 2 h. The sample processing was conducted according to the manufacturer’s instructions. After the final RNA pull-down, biotinylated RNAs were separated from the beads in TRIzol™ reagent. Sample quality control/fragment size distribution was assessed using the Bioanalyzer system (Agilent). The concentration of samples was measured using the Quantus™ Fluorometer. Isolated RNA samples with an RNA Integrity Number (RIN) higher than 9 were validated for further sequencing analysis. Construction of cDNA libraries from RNA samples was done using the Collibri™ 3′ mRNA Library Prep Kit (Invitrogen). Samples were sequenced on a NextSeq 550 (Illumina) system using a NextSeq 500/550 Mid Output v2.5 (75 Cycles) cartridge (Single-End). The number of samples per cartridge was arranged in a way to provide a minimum of 20 M raw reads per 3′mRNAseq sample.

### dCAS9-mediated gene expression

Vectors for dCAS9 and sgRNAs containing MS2 binding sites are based on previous reports^60,82^. Sequences encoding MS2 and 3 Flag-tags were obtained as gene blocks [Integrated DNA Technologies (IDT)] and cloned into a CMV-driven vector containing a Gateway cloning cassette. Sequences for ASH2L and mutants were recombined into this vector. HEK293 cells were co-transfected with plasmids expressing dCAS9, sgRNAs targeting the *HBG1* promoter^60^, and MS2 and MS2-ASH2L fusion proteins expressing vectors. RNA was harvested and reverse transcribed as described above. *HBG1* expression was analyzed using RT-qPCR assays with QuantiTect primers. For control, *β-glucuronidase* was measured (forward: CTCATTTGGAATTTTGCCGATT; reverse: CCGAGTGAAGATCCCCTTTTTA).

### Bioinformatics

#### Statistical analysis

When multiple samples were compared, one-way-ANOVA with a posthoc Dunnett’s test was applied with the untreated samples serving as control. The statistically different values are indicated. Non-significant differences in these multi-sample analyses are not indicated.

#### Read trimming

The raw reads of all sequencing experiments (ChIP-seq, ATAC-seq, RNA-seq and Click-it) were trimmed using Trim_Galore (https://www.bioinformatics.babraham.ac.uk/projects/trim_galore/).

#### Reference genome

The reference genome used here was the mouse reference genome mm10 (GRCm38.p6; http://ftp.ebi.ac.uk/pub/databases/gencode/Gencode_mouse/release_M25/).

#### Read alignment

BWA was used to align the trimmed reads from ChIP-seq and ATAC-seq to mm10 (Ref^83^). The Trimmed reads from RNA-seq and Click-it were aligned to mm10 using STAR^84^. Samtools were used to generate the sorted Bam files after filtering the unusable reads as recommended by Encode^83^. Quality controls for both ChIP-seq and ATAC-seq were done as recommended by Encode. The replicates were merged using Picard MergeSamFiles (https://github.com/broadinstitute/picard/blob/master/src/main/java/picard/sam/MergeSamFiles.java).

#### Peak calling and featureCounts

Narrow peaks (ChIP-seq (H3K4me3, H3K27ac, ASH2L) and ATAC-seq) and broad peaks (ChIP-seq (H3K4me1, H3K27me3)) were called using Macs^85^. In RNA-seq and Click-it, the reads aligned to the mm10 genome were assigned to the known annotated mm10 genes using featureCounts^86^. The counts obtained from featureCounts were normalized using the External RNA Control Consortium (ERCC, Invitrogen) spike-in.

#### Data visualization

DeepTools were used to generate both heatmaps and plotprofiles showing the normalized signal (normalized using CPM (count per Million)) for the ChIP-seq or ATAC-seq experiments^87^. This was centered either at TSS of all annotated transcripts in mm10 or at enhancers we identified during this work. MA plots were generated using the ggmaplot function of ggplot2 package in Rstudio^88^. The apeglm method for log2 fold change shrinkage was applied for the MA plots^89^. The sampleDistMatrix function of pheatmap package in Rstudio was used to generate the heatmaps (Raivo Kolde (2019). pheatmap: Pretty Heatmaps. R package version 1.0.12. http://CRAN.R-project.org/package=pheatmap). IGV (Integrative Genomics Viewer, https://software.broadinstitute.org/software/igv/) was used to visualize the normalized BigWig files and to evaluate the results.

The genes that significantly lost H3K4me3 signal after 2 h of FKBP-HA2-ASH2L loss were analyzed for the associated Gene Ontology (GO) terms for biological processes using the enrichGO function of the package clusterProfiler in Rstudio^90^. Dot plot visualizing the results was generated using the dotplot function of ggplot2 package in Rstudio^88^.

#### Defining putative enhancers

Enhancers were defined by positivity for H3K4me1, H3K27ac and ATAC signal in the control samples excluding the overlap with ± 1 kb of TSS of the known mm10 promoters.

#### Promoters categories (fast/slow and high/medium/low)

The promoters that significantly lost H3K4me3 (q < 0,05; logFC > 0.58) were categorized as fast and slow as follows: fast (499 promoters) are all promoters that showed a significant loss of H3K4me3 signals after 2 h, slow (788 promoters) are all promoters that showed a significant loss of H3K4me3 signals only after 16 h (excluding promoters significantly losing H3K4me3 signal after 30 min, 2 h, 4 h or 8 h). In addition, all identified H3K4me3 across all samples (25431 sites) were divided into three equal categories according to the signal intensity: high, medium and low (8477 each).

#### Intersection

The overlap between different comparisons in the same sequencing experiment and between different experiments were done using BEDTools^91^.

#### Normalization

ChIP-seq samples were normalized to the lowest coverage in each experiment. In ATAC-seq, the data was normalized using DESeq2^92^.

#### Differential analysis

Differential analysis for each ChIP-seq, ATAC-seq, RNA-seq and Click-it experiment was carried out using DESeq2 to identify the significantly changed sites.

#### Annotation

The significantly changed sites were then annotated using Homer (http://homer.ucsd.edu/homer/ngs/annotation.html). The information after annotation (distance to the nearest promoter provided by Homer) was used to identify the distance to TSS. Coordinates of CGI in mm10 were obtained from UCSC (https://hgdownload.soe.ucsc.edu/goldenPath/mm10/database/cpgIslandExt.txt.gz).

#### Transcription factor footprinting

In ATAC-seq, transcription factor (TF)-footprinting analysis was performed using RGT-HINT^80^.

#### Codes

Sections of the codes from nf-core were modified and used for ChIP-seq (https://nf-co.re/chipseq/) and ATAC-seq (https://nf-co.re/atacseq) analyses.

### Supplementary Information

Supplementary Information.

## Data Availability

All sequencing data are available in NCBI’s Gene Expression Omnibus (GEO; Ref^93^) as SuperSeries under accession number GSE241174 (https://www.ncbi.nlm.nih.gov/geo/query/acc.cgi?acc=GSE241174). This SuperSeries is composed of the following sub-series: 1. Accession number GSE240987 for RNA-seq. 2. Accession number GSE241001 for ASH2L ChIP-seq. 3.Accession number GSE240994 for H3K4me3 ChIP-seq. 4. Accession number GSE240992 for H3K4me1 ChIP-seq. 5. Accession number GSE240990 for H3K27ac ChIP-seq. 6. Accession numbers GSE240999 and GSE241000 for H3K27me3 ChIP-seq. 7. Accession number GSE241169 for ATAC-seq. 8. Accession number GSE239789 for Click-it-seq.
