# Supplementary material for: Sequential deregulation of histone marks, chromatin accessibility and gene expression in response to PROTAC-induced degradation of ASH2L
Source: Sci Rep. 2023 Dec 19;13:22565. doi: 10.1038/s41598-023-49284-x (PMC10730889; doi:10.1038/s41598-023-49284-x)
Supplement: Supplementary file 1 — Supplementary Information. [file 41598_2023_49284_MOESM1_ESM.pdf]

Supplementary information

Sequential deregulation of histone marks, chromatin accessibility and gene expression in response to PROTAC-induced degradation of ASH2L

Mirna Barsoum[1,2], Roksaneh Sayadi-Boroujeni[1,2,3], Alexander T. Stenzel[1,4], Philip Bussmann[1], Juliane Lüscher-Firzlaff[1,5], and Bernhard Lüscher[1]

[1]Institute of Biochemistry and Molecular Biology, Faculty of Medicine, RWTH Aachen University, Pauwelsstrasse 30, 52074 Aachen, Germany
[2]Contributed equally
[3]Present address: Bayer AG, Crop Science Division, R&D, Pest Control, 40789 Monheim am Rhein, Germany
[4]Present address: Institute of Human Genetics, Faculty of Medicine, University of Bonn, Venusberg-Campus 1, 53127 Bonn
[5]Retired

Correspondence to M.B. (mirna.barsoum@rwth-aachen.de) and B.L. (luescher@rwth-aachen.de)

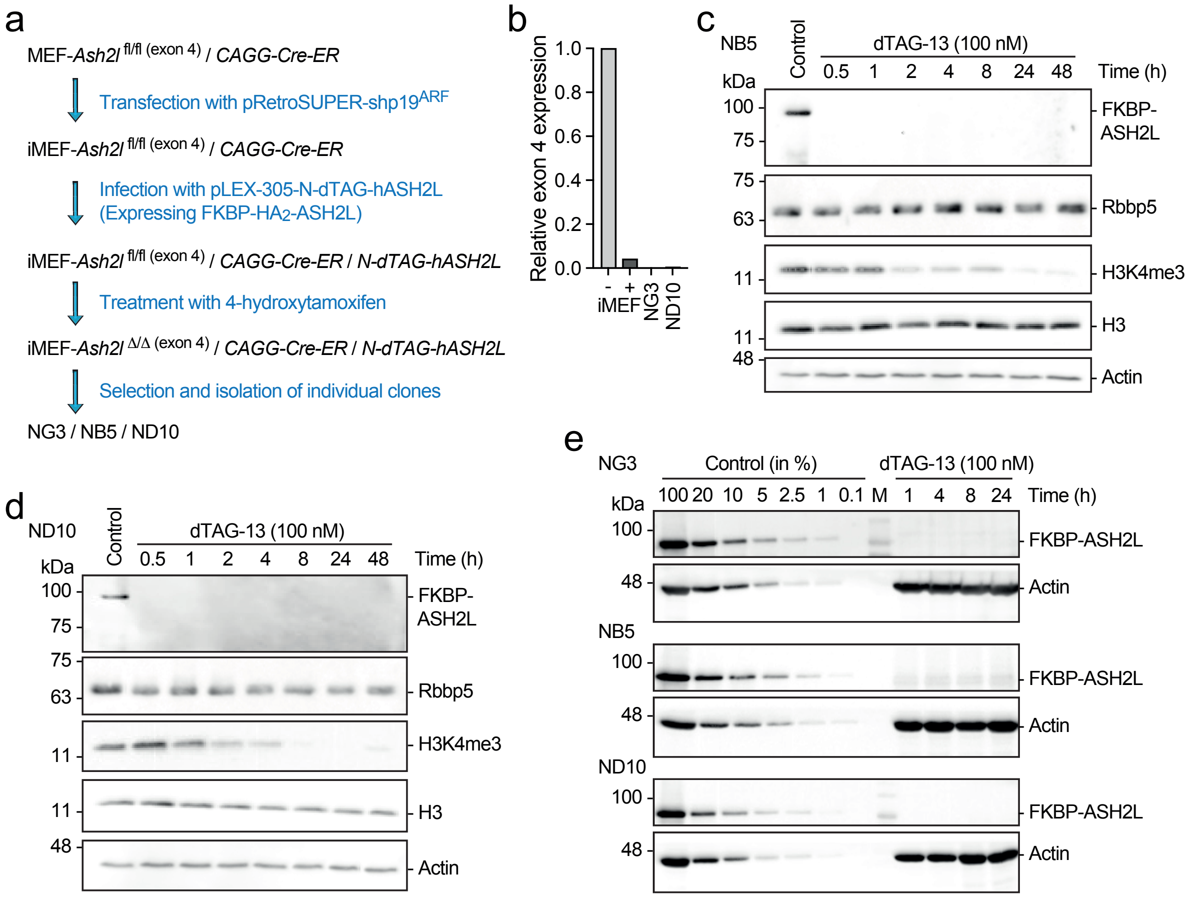

Supplementary Figure S1 (supporting Figures 1 and 2)

(a) Schematic summary of the steps employed to generate the mouse embryo fibroblast clones that are dependent on the FKBP-HA_2-ASH2L fusion protein. The *CAGG-Cre-ER* transgene contains the CMV-IE enhancer/chicken β-actin/rabbit β-globin hybrid promoter and encodes for a CreER fusion protein{Hayashi, 2002 #1151}.

(b) RT-qPCR analysis of exon 4 of *Ash2l* from iMEF cells treated ±hydroxytamoxifen (-/+) or from the NG3 and ND10 cell clones.

(c-e) FKBP-HA_2-ASH2L expressing cells were treated with dTAG-13. The indicated proteins were identified on Western blots, the cell clones analyzed are indicated.

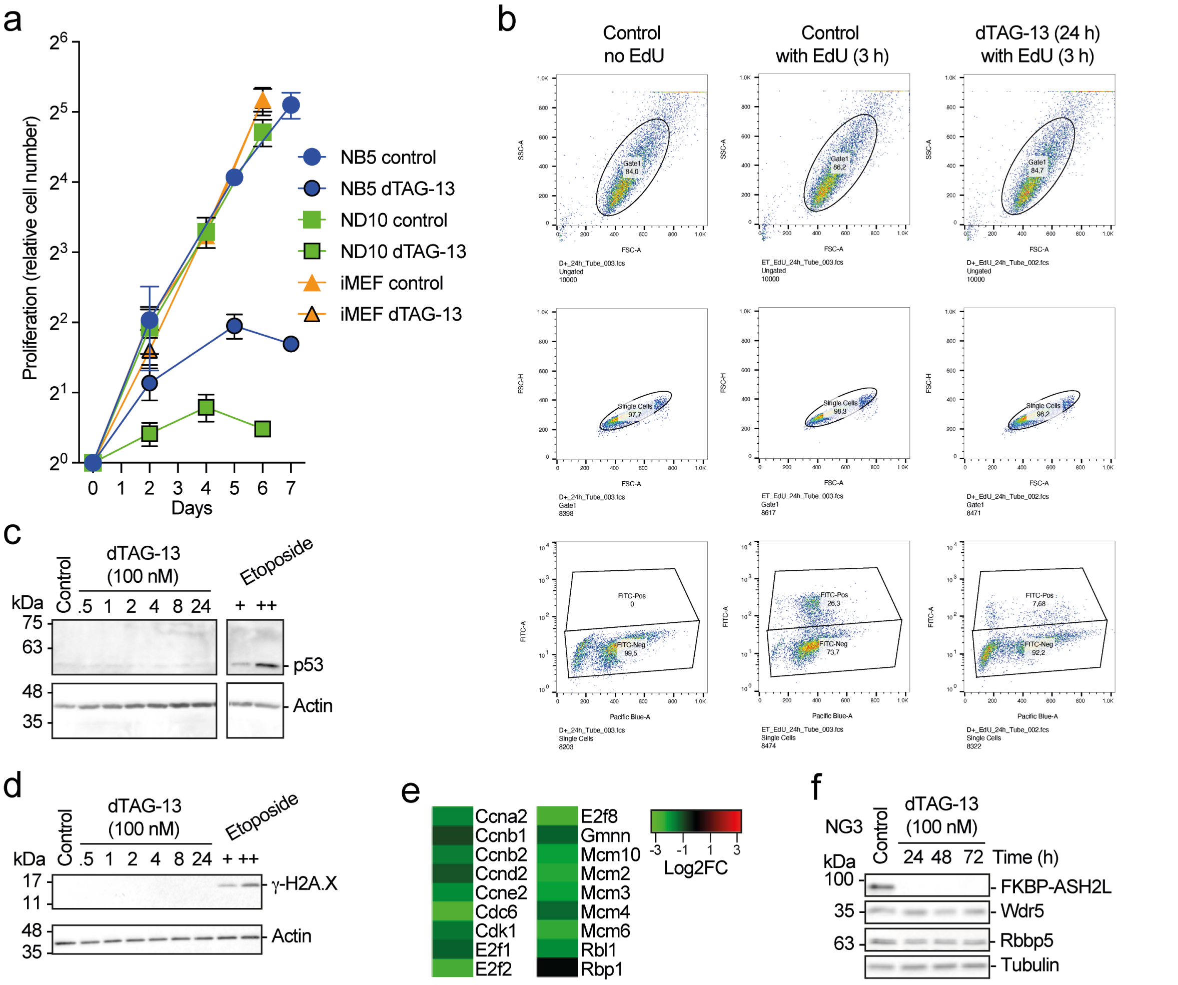

Supplementary Figure S2 (supporting Figures 1 and 2)

(a) Clones expressing FKBP-HA$_2$-ASH2L fusion proteins (NB5 and ND10) and control cells (iMEF) were treated with or without dTAG13 from day 0. Cells were counted at the indicated time points. Measurement were in triplicates with three biological replicates. Indicated are relative mean values ±SEM. The control iMEF cells are identical to the control cells in Fig. 1d.

(b) Examples of the flow cytometry analysis of cells treated ±dTAG-13 for 24 hours and treated with EdU for the final 3 hours. The top row shows the gate used for further analysis. The middle row defines single cells. The bottom row shows the selection of FITC positive and negative cells.

(c and d) NG3 cells were treated with dTAG-13 as indicated. For control, cells were treated with etoposide (+ and ++ indicate 100 and 400 µM, respectively) for 4 hrs. p53 and γ-H2AX were stained as indicated. Actin staining served as control.

(e) Down-regulated genes, which encode cell cycle regulators and replication factors after dTAG-13 treatment for 24 hrs (from Supplementary Table S1).

(f) Analysis of the WRAD components Wdr5 and Rbbp5 upon loss of FKBP-HA$_2$-ASH2L. dTAG-13 (100 nM) treatment was for the times indicated. Tubulin is shown for control.

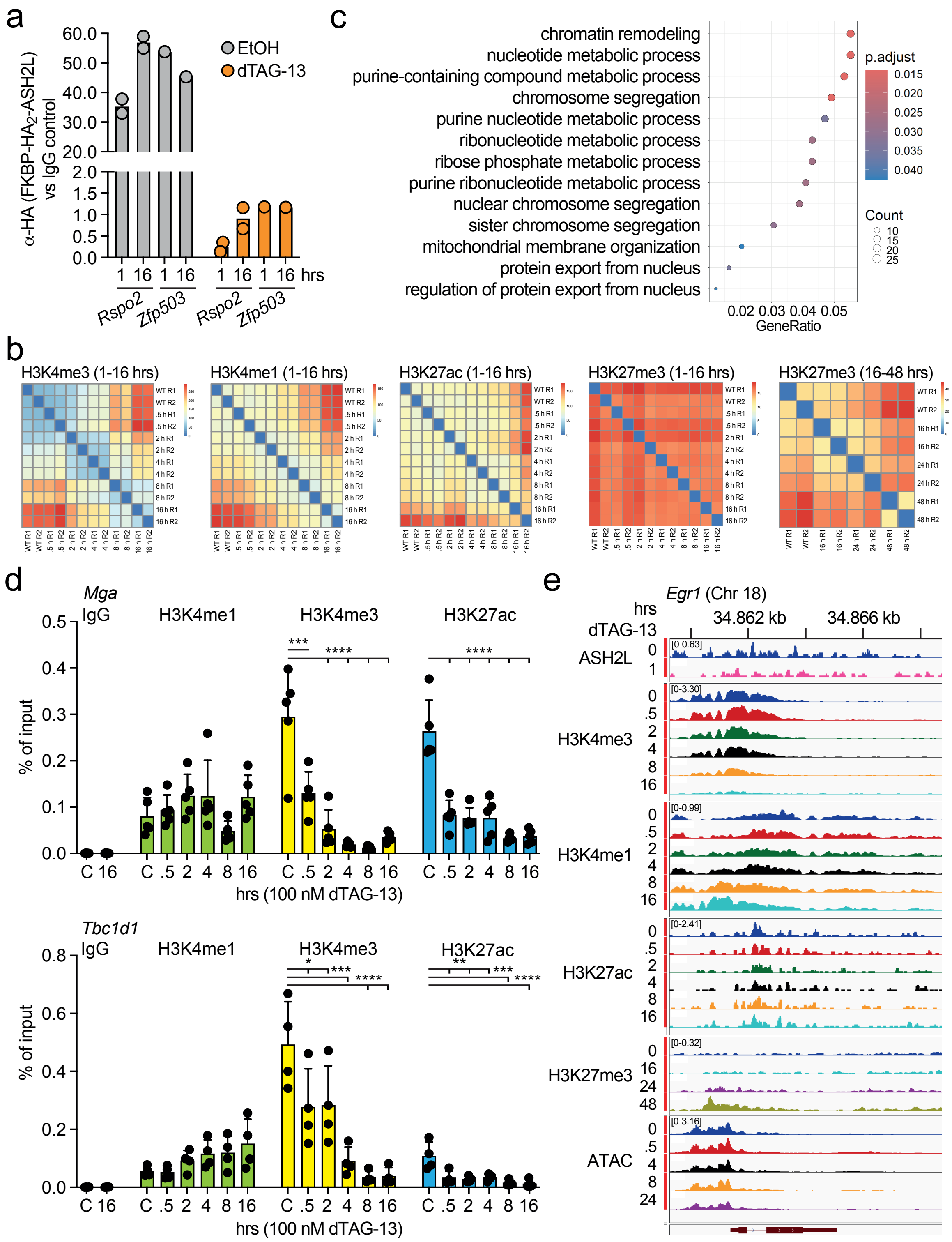

Supplementary Figure S3 (supporting Figure 3)

(a) Cells were treated with or without dTAG-13 (100 nM) for 1 or 16 hrs. ChIP-qPCR were performed using either HA-specific antibodies to immunoprecipitate FKBP-HA$_2$-ASH2L or control antibodies. Mean values of two replicates are displayed.

(b) Heatmaps showing sample-to-sample distances comparing all ChIP-seq samples of the indicated histone marks for both biological replicates to each other.

(c) Biological pathways associated with the genes associated with the 663 downregulated H3K4me3 sites after 2 hours upon loss of ASH2L (GO “biological process”, q value <0.05).

(d) ChIP-qPCR analyses of selected promoters for the indicated histone marks. Indicated are mean values ±SD of 4 to 5 measurements. (* $p \leq 0.05$; *** ≤ 0.001; **** $p \leq 0.0001$)
(e) Screen shots of integrative genomics viewer (IGV) of the *Egr1* promoter region showing the normalized BigWig tracks (normalized using counts per Million) of ChIP-seq experiments of the indicated histone marks and of ASH2L. Additionally, the chromatin accessibility is summarized as measured by ATAC-seq at the indicated timepoints after dTAG-13 treatment.

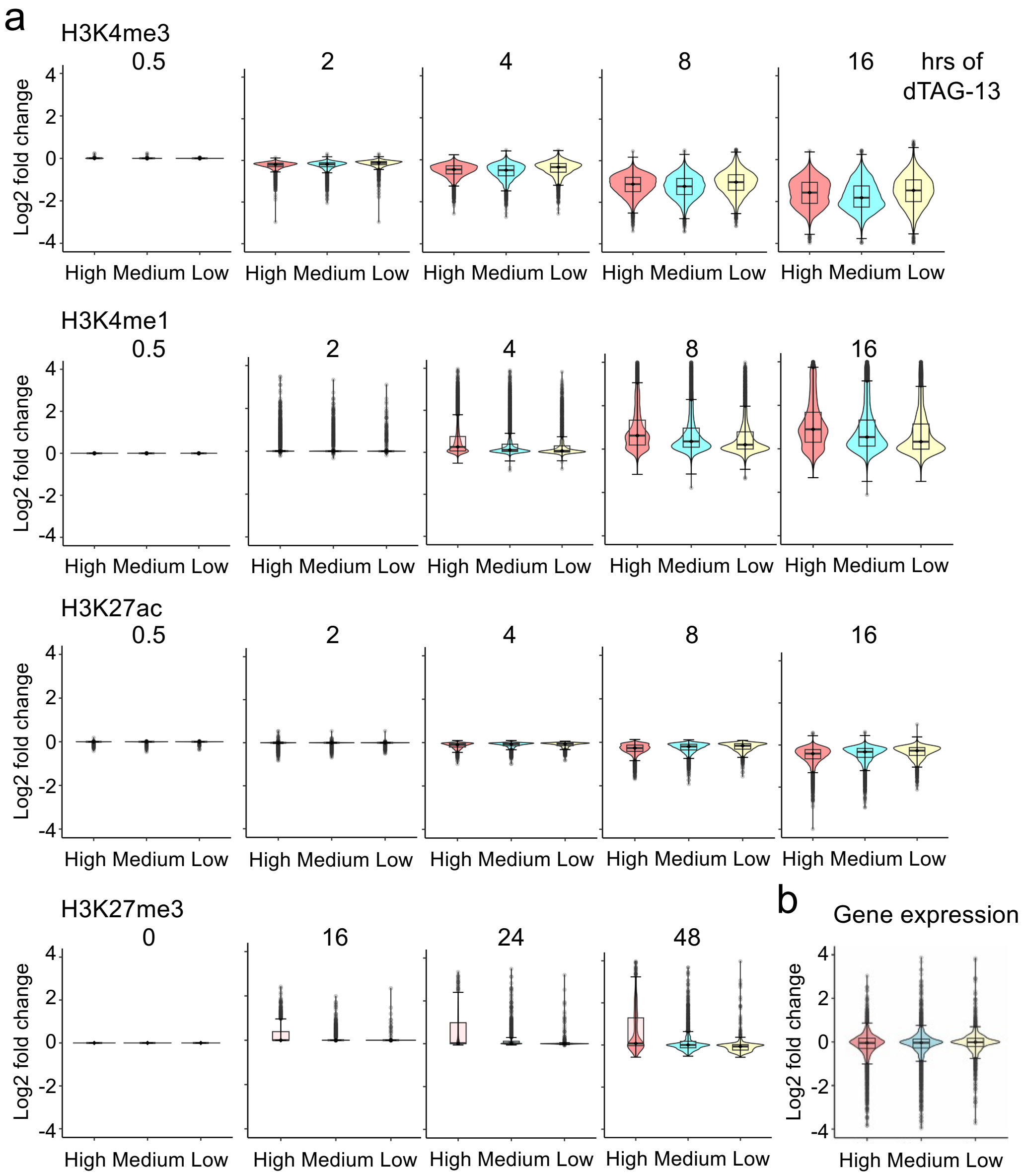

Supplementary Figure S4 (supporting Figure 4)

(a) H3K4me3 binding sites (25431) were divided into three equal groups (high, medium and low, 8477 each). A window of ±1 kb around transcriptional start sites of all promoters was considered. The total number of binding sites within this window at promoters in the categories high, medium and low is 7937, 5413 and 1955, respectively. The log2 fold changes of signals of the indicated histone marks at the promoters of these three classes are displayed. For H3K27me3 the 2, 4 and 8 hour time points are not shown as no changes were observed.

(b) Gene expression as determined by RNA-seq after 24 hours of dTAG-13 treatment was linked to promoters with high, medium and low H3K4me3 in control cells.

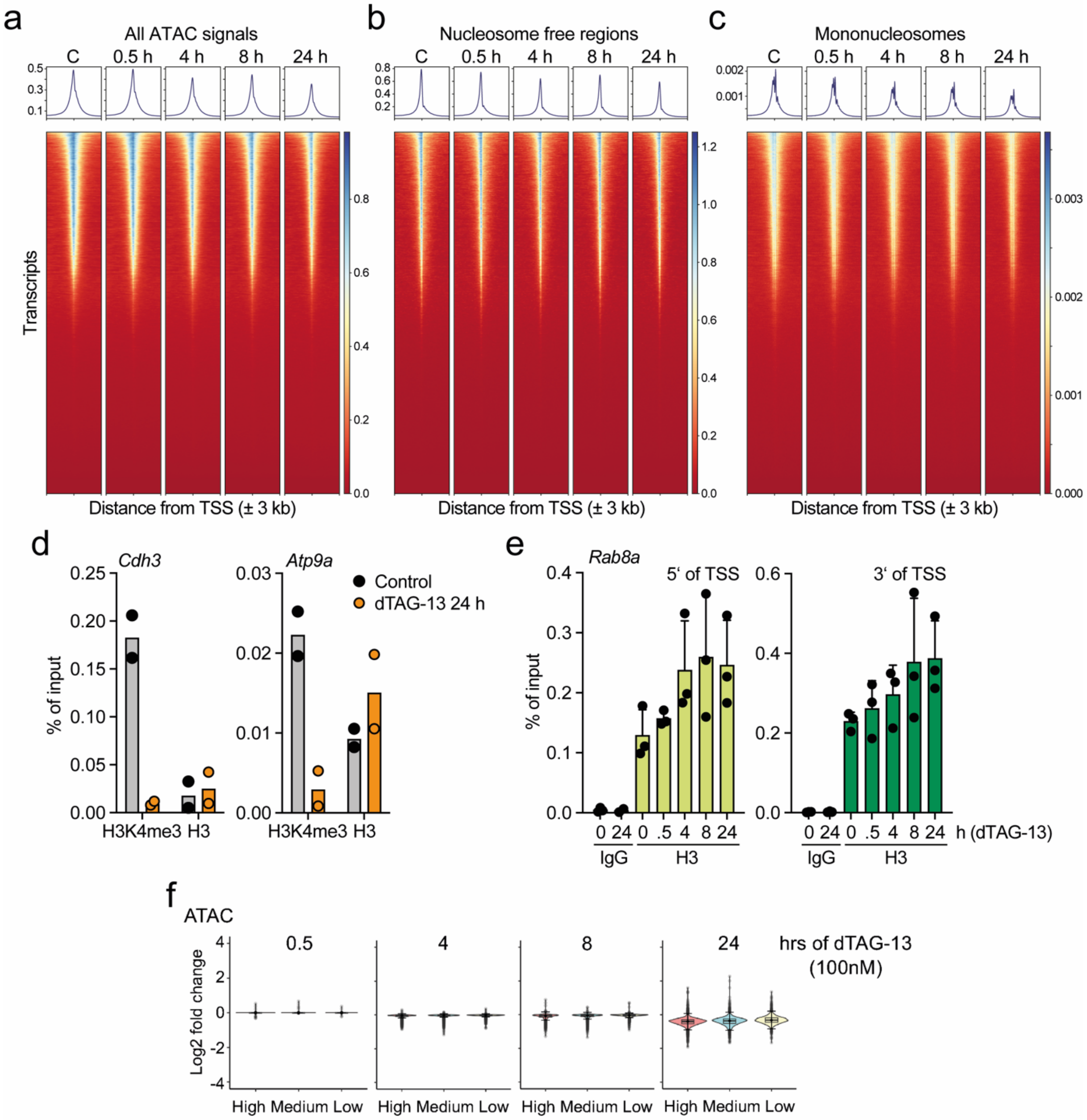

Supplementary Figure S5 (supporting Figure 6)

(a-c) Heatmaps and plot profiles generated using DeepTools showing the normalized ATAC-seq signals in response to dTAG-13 treatment at ±1 kb of transcriptional start sites (TSSs) of all annotated transcripts in mm10 (normalized using counts per Million). Nucleosome-free and mono-nucleosomes refer to ATAC-seq fragments that are smaller than 120 bp and between 130 and 200 bp, respectively.

(d and e) ChIP-qPCR measurements of H3K4me3 and histone H3. IgG was used for control. Between 2 and 3 experiments are displayed with mean values ±SD.

(f) Three equal groups of H3K4me3 signals were evaluated for being promoter associated (±1 kb of TSSs). This resulted in 7937, 5413 and 1955 sites in the three groups high, medium and low, respectively. The log2 fold changes of ATAC-seq signals at the promoters of these three groups are displayed.

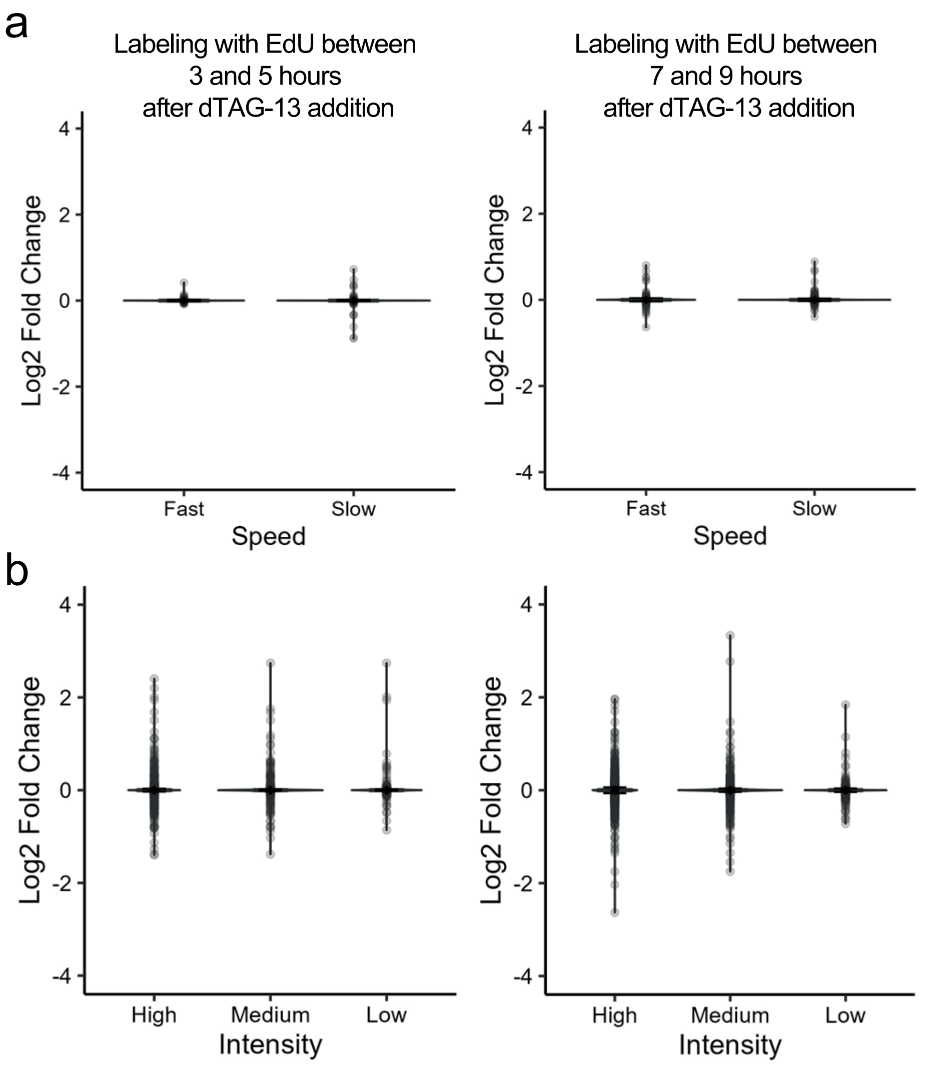

Supplementary Figure S6 (supporting Figure 8)

Cells were labeled with dTAG-13 and EdU as indicated. The significantly deregulated genes (see Fig. 8a, Supplementary Table S11) were analyzed for their association with promoters that lost H3K4me3 fast or slow (panel a). Moreover, these genes were assessed for their link to the three equal classes of promoters with high, medium and low levels of H3K4me3.

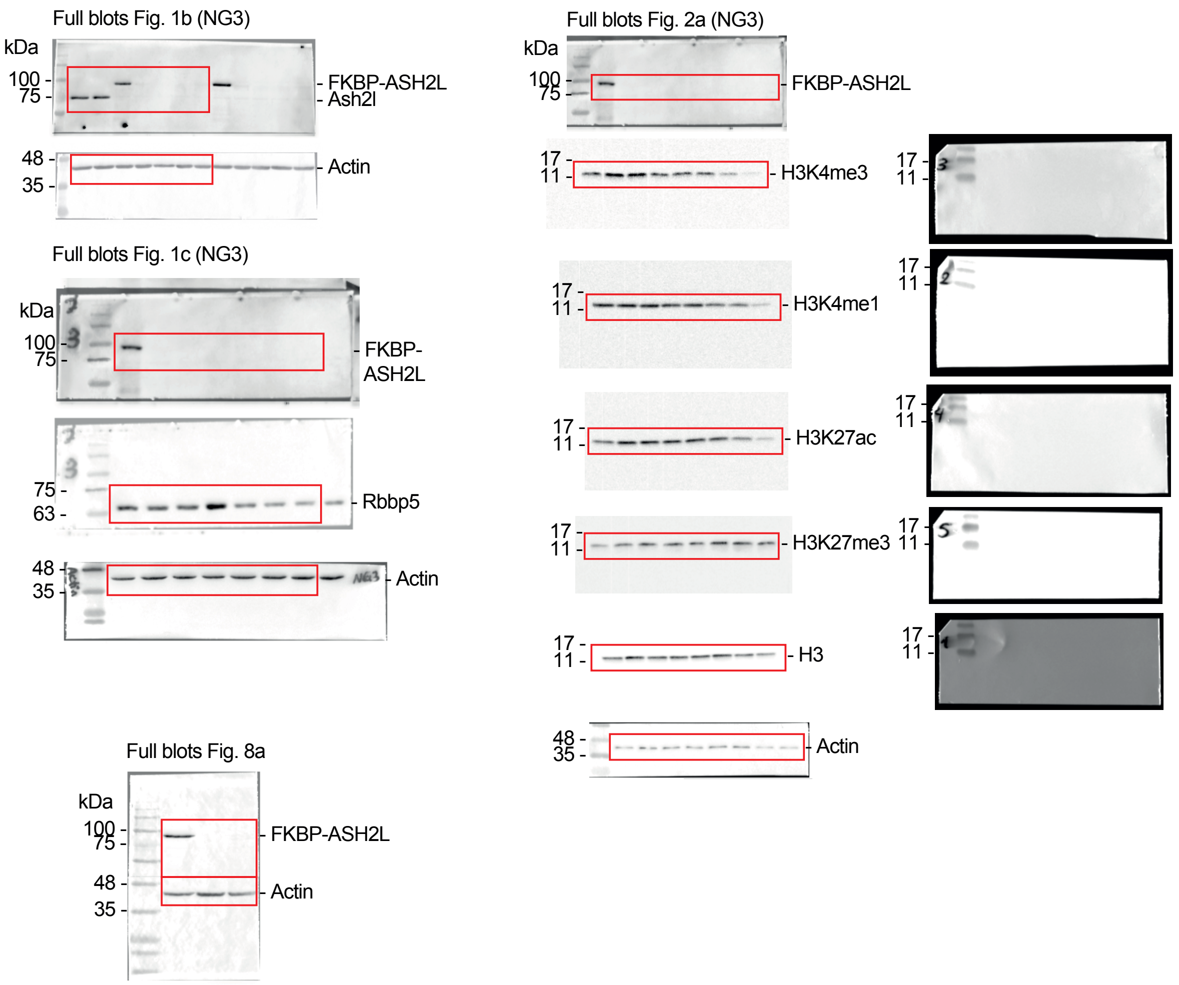

Supplementary Figure S7 (supporting Figures 1, 2 and 8)

All the blots were cut prior to hybridization with antibodies, typically the different parts were probed with distinct antibodies. The edges of some blots are difficult to see because of the signal strength. For these the membranes pictured with reflected light are shown.

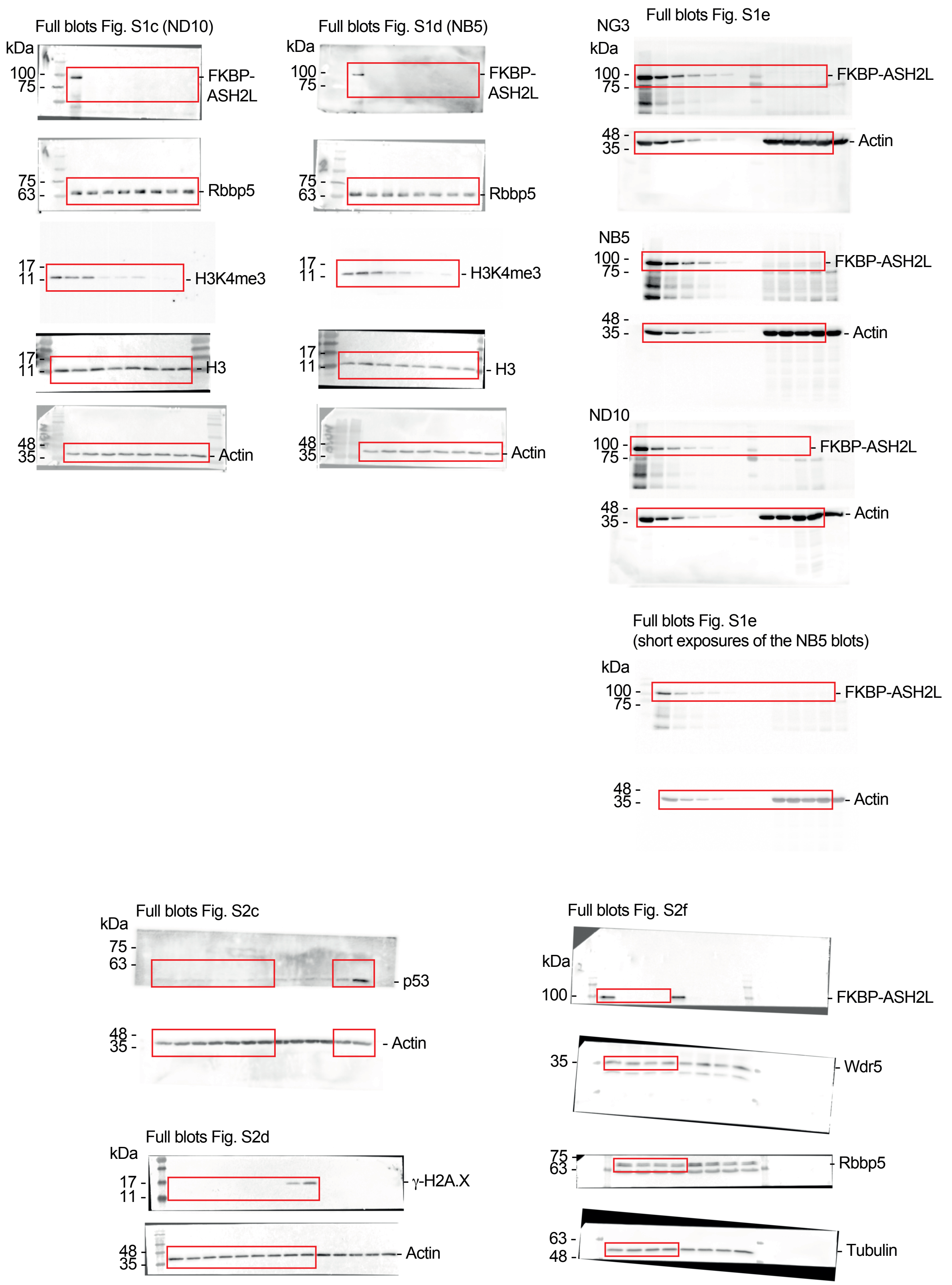

Supplementary Figure S8 (supporting Supplementary Figures 1 und 2)
All the blots were cut prior to hybridization with antibodies, typically the different parts were probed with distinct antibodies.

Supplementary Tables

All sequencing data are available in NCBI's Gene Expression Omnibus as SuperSeries under accession number GSE241174:
https://www.ncbi.nlm.nih.gov/geo/query/acc.cgi?acc=GSE241174.

Table S1 List of significantly changed genes after 24h compared to the WT (RNA-seq); available in GEO under accession number GSE240987

Table S2a All identified Ash2l peaks across all the samples; available in GEO under accession number GSE24100.

1**Table S2b** List of significantly changed Ash2l binding sites after 1h compared to the WT (ChIP-seq Ash2l) ; available in GEO under accession number GSE241001.

Table S3a All identified H3K4me3 peaks across all the samples; available in GEO under accession number GSE240994.

Table S3b List of significantly changed H3K4me3 binding sites in each timepoint compared to the WT (ChIP-seq H3K4me3); available in GEO under accession number GSE240994.

Table S4a All identified H3K4me1 peaks across all the samples; available in GEO under accession number GSE240992.

Table S4b List of significantly changed H3K4me1 binding sites in each timepoint compared to the WT (ChIP-seq H3K4me1); available in GEO under accession number GSE240992.

Table S5a All identified H3K27ac peaks across all the samples; available in GEO under accession number GSE240990.

Table S5b List of significantly changed H3K27ac binding sites in each timepoint compared to the WT (ChIP-seq H3K27ac); available in GEO under accession number GSE240990.

Table S6a All identified H3K27me3 peaks across all the samples (ChIP-seq H3K27me3_First experiment); available in GEO under accession number GSE240999.

Table S6b List of significantly changed H3K27me3 binding sites in each timepoint compared to the WT (ChIP-seq H3K27me3_First experiment); available in GEO under accession number GSE240999.

Table S7a All identified H3K27me3 peaks across all the samples (ChIP-seq H3K27me3_Second experiment); available in GEO under accession number GSE241000.

Table S7b List of significantly changed H3K27me3 binding sites in each timepoint compared to the WT (ChIP-seq H3K27me3_Second experiment); available in GEO under accession number GSE241000.

Table S8a All identified peaks across all the samples (ATAC-seq); available in GEO under accession number GSE241169.

Table S8b List of sites significantly changed in accessibility in each timepoint compared to the WT (ATAC-seq); available in GEO under accession number GSE241169.

Table S9 List of all identified putative Enhancers; available in GEO under accession number GSE240994.

Table S10 List of significantly changed TF (ATAC-seq; differential analysis using RGT-Hint); available in GEO under accession number GSE241169.

Table S11 List of significantly changed genes after 4h or 8h compared to the WT (Nascent RNA; Click-it); available in GEO under accession number GSE239789.
